# Development of Yellow-to-Orange Photoluminescence Molecules Based on Alterations in the Donor Units of Fluorinated Tolanes

**DOI:** 10.3390/molecules27185782

**Published:** 2022-09-07

**Authors:** Shigeyuki Yamada, Kazuki Kobayashi, Tsutomu Konno

**Affiliations:** Faculty of Molecular Chemistry and Engineering, Kyoto Institute of Technology, Matsugasaki, Sakyo-ku, Kyoto 606-8585, Japan

**Keywords:** aggregation-induced emission (AIE), donor-π-acceptor, fluorine, fluorescence, hydrogen bonds, intramolecular charge transfer (ICT), tolane

## Abstract

Since the aggregation-induced emission (AIE) phenomenon was first reported by Tang et al., much effort has been devoted to the development of solid-state luminescent molecules by chemists worldwide. Our group successfully developed fluorinated tolanes as novel compact π-conjugated luminophores with blue photoluminescence (PL) in the crystalline state. Moreover, we reported the yellow-green PL molecules based on their electron-density distributions. In the present study, we designed and synthesized fluorinated tolanes with various amine-based donors and evaluated their photophysical properties. The carbazole-substituted fluorinated tolane exhibited strong PL in the solution state, whereas piperidine- or phenothiazine-substituted fluorinated tolanes showed a dramatic decrease in PL efficiency. Notably, fluorinated tolanes with piperidine or phenothiazine substituents displayed yellow-to-orange PL in the crystalline state; this may have occurred because these tolanes exhibited tightly packed structures formed by intermolecular interactions, such as H···F hydrogen bonds, which suppressed the non-radiative deactivation process. Moreover, fluorinated tolanes with amine-based donors exhibited AIE characteristics. We believe that these yellow-to-orange solid PL molecules can contribute to the development of new solid luminescent materials.

## 1. Introduction

Solid-state luminescent materials used in lighting and luminescent devices are recognized as essential materials in our lives. Although many luminescent molecules have been reported so far [1,2,3,4], the development of solid-state luminescent molecules has been considerably delayed because of the special molecular design required [5,6,7]. Since Tang et al. first reported the aggregation-induced emission (AIE) [8,9,10] and crystallization-induced emission (CIE) phenomena [11,12,13], solid-state luminescent molecules have gradually appeared, and their further development is still ongoing.

**Figure 1:**
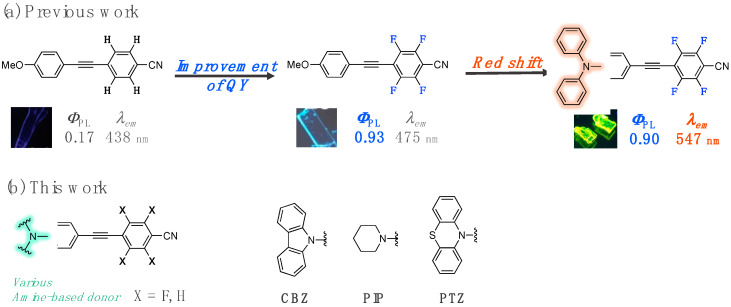
(**a**) Changes in the photoluminescence (PL) behavior of tolanes based on structural modulations. (**b**) Chemical structure of a fluorinated tolane with an amine-based donor used in this study.

Over the last few years, our group has focused on the development of fluorinated organic luminophores [14,15,16,17] because the incorporation of fluorine atoms into organic molecules often provides them with unique characteristics, owing to the special properties of the fluorine atom. For example, methoxy-substituted donor-π-acceptor (D-π-A)-type fluorinated tolane exhibited intense blue photoluminescence (PL) in the crystalline state; however, its non-fluorinated counterpart displayed very weak PL, which was caused by its immediate internal conversion from the emissive ππ* excited state to the non-emissive πσ* excited state at ambient temperature (Figure 1a) [14]. Furthermore, replacing the methoxy group with a diphenylamino group in the fluorinated tolane molecule led to a significant change in the PL color from blue to yellow-green, which was due to its high electron density distribution (Figure 1a) [17].

Among solid-state luminescent molecules, luminophores showing orange-to-red PL have recently attracted significant attention because of their application in full-color light-emitting diodes [18,19]. However, it is well known that PL wavelength (*λ*_PL_) and PL efficiency (*Φ*_PL_) are inversely proportional to each other; many strategies, such as molecular recognition [20], molecular assembly [21,22], and co-crystallization [23], have been utilized to increase the PL wavelength. Owing to these molecular designs, many luminophores with solid-state yellow-to-red fluorescence and phosphorescence have been reported to date [24,25,26,27].

To develop solid-state PL molecules exhibiting long-wavelength PL with a compact π-conjugated structure, we designed novel compact π-conjugated molecules with a long-wavelength PL based by appropriately controlling their electron density and molecular orbital distributions [14,17]. Therefore, in this study, we synthesized a series of D-π-A-type fluorinated tolanes with amine-based donors, viz., **CBZ-F** with a carbazole unit, **PIP-F** with a piperidine unit, and **PTZ-F** with a phenothiazine unit, and evaluated their photophysical behavior (Figure 1b). The presence of the amine-based donors can control the molecular orbital and electron-density distributions of the tolanes. To determine the effect of the fluorine atoms in the tolane scaffold, non-fluorinated compounds, **CBZ-H**, **PIP-H**, and **PTZ-H**, were synthesized, and their photophysical behavior was studied in detail. In this article, the photophysical behavior of the aforementioned six analogs is described and discussed based on their molecular aggregated structures.

## 2. Results and Discussion

### 2.1. Synthesis

**Figure 2:**
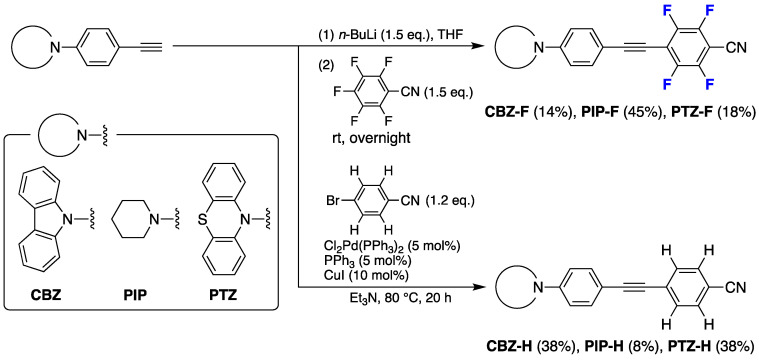
Synthetic procedure for a series of fluorinated and non-fluorinated tolanes with amine-based donors.

Initially, we synthesized a series of fluorinated tolanes, **CBZ-F**, **PIP-F**, and **PTZ-F**, and their non-fluorinated counterparts, **CBZ-H**, **PIP-H**, and **PTZ-H**, according to a previously reported synthetic procedure [17]. The synthetic procedure is illustrated in Figure 2.

Lithium 4-(carbazol-9-yl)phenylacetylide, prepared from 4-(carbazol-9-yl)phenylacetylene and *n*-butyllithium, reacted with pentafluorobenzonitrile in THF at room temperature for 18 h; the resulting nucleophilic aromatic substitution (S_N_Ar) reaction afforded **CBZ-F** in 14% isolated yield. The other analogs, **PIP-F** and **PTZ-F**, were synthesized in 45% and 18% isolated yields, respectively, in a similar manner. The non-fluorinated compounds, **CBZ-H**, **PIP-H**, and **PTZ-H**, were obtained in 8–38% yield via the palladium(0)-catalyzed Sonogashira cross-coupling reaction of 4-bromobenzonitrile with 4-amino substituted phenylacetylene. All synthesized compounds were purified by column chromatography and subsequently recrystallized. The structures of these molecules were confirmed by ^1^H nuclear magnetic resonance (NMR), ^13^C NMR, and ^19^F NMR spectroscopy. Moreover, their infrared spectra and high-resolution mass spectra (HRMS) were obtained. These spectra proved that the target molecules were sufficient to assess the photophysical behavior (Figures S1–S15 in the Supplementary Information).

### 2.2. Photophysical Behavior in Solution State

**Figure 3:**
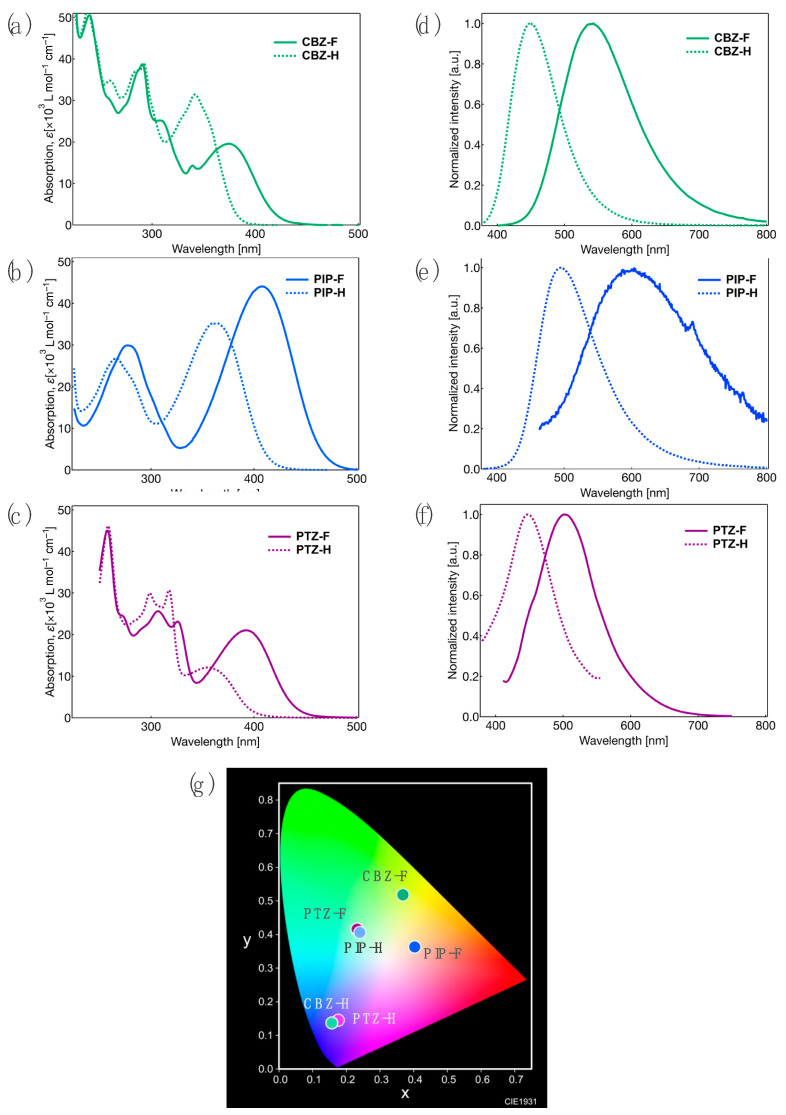
UV–vis absorption spectra of (**a**) **CBZ-F** and **CBZ-H**, (**b**) **PIP-F** and **PIP-H**, and (**c**) **PTZ-F** and **PTZ-H** in CH_2_Cl_2_. PL spectra of (**d**) **CBZ-F** and **CBZ-H**, (**e**) **PIP-F** and **PIP-H**, and (**f**) **PTZ-F** and **PTZ-H** in CH_2_Cl_2_. Concentration: 1.0 × 10^−5^ mol L^−1^, (**g**) CIE color diagram of all derivatives.

The ultraviolet–visible (UV–vis) and PL spectra of **CBZ-F**, **PIP-F**, and **PTZ-F** and their non-fluorinated counterparts, **CBZ-H**, **PIP-H**, and **PTZ-H**, were obtained using their solutions in dichloromethane (CH_2_Cl_2_). Figure 3 shows the UV–vis and PL spectra of the tolanes, and their related photophysical data are summarized in Table 1.

In the absorption spectra of **CBZ-F** and **CBZ-H** (Figure 3a), the absorption bands in the short-wavelength region below 300 nm were found to be almost identical. However, a slight absorption band shift was observed in the long-wavelength region above 300 nm. The maximum absorption wavelength (*λ*_abs_) for **CBZ-F** was 375 nm, which was red-shifted by 33 nm compared to that of **CBZ-H**. The **PIP** and **PTZ** analogs also exhibited similar absorption behavior. Both fluorinated and non-fluorinated compounds showed an identical spectrum in the short-wavelength region below 300 nm. However, the fluorinated compounds (**PIP-F** and **PTZ-F**) exhibited a red-shifted *λ*_abs_ (by 37–45 nm) for the absorption bands observed in the long-wavelength region (Figure 3b,c).

**Figure 4:**
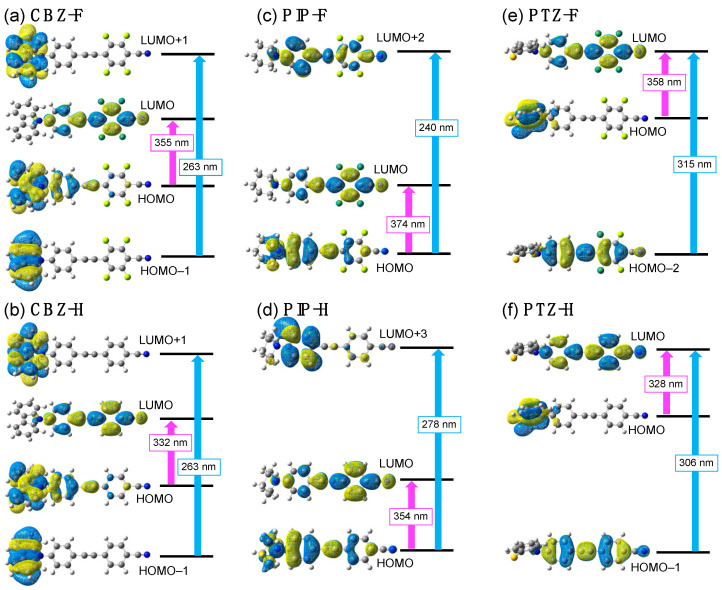
Molecular orbital distributions including theoretical electronic transition calculated using TD-DFT for (**a**) **CBZ-F**, (**b**) **CBZ-H**, (**c**) **PIP-F**, (**d**) **PIP-H**, (**e**) **PTZ-F**, and (**f**) **PTZ-H**.

To investigate the theoretical electronic transitions of the tolanes, theoretical calculations were performed using the Gaussian 16 program set [28]. Herein, the calculations were conducted using time-dependent density functional theory (TD-DFT) at the M06-2X/6-31 + G(d,p) [29] level of theory with a conductor-like polarizable continuum model (CPCM) [30] for CH_2_Cl_2_, which was used as an implicit solvation model. **CBZ-F**, **PIP-F**, **CBZ-H**, and **PIP-H** showed two allowed electronic transitions (Figure 4, Table 2).

In the case of **CBZ-F** and **CBZ-H**, the electronic transition from the highest occupied molecular orbital (HOMO) to the lowest unoccupied molecular orbital (LUMO) was assigned to the long-wavelength absorption band, which was calculated as the electronic transition from HOMO−1 to LUMO+1. The HOMO lobe is mainly localized over the electron-rich aromatic ring substituted with a carbazole moiety, while the LUMO is localized over the electron-deficient fluorinated aromatic ring. The HOMO → LUMO transition with a low transition energy is considered as a charge transfer (CT) transition, which probably causes the red-shift of *λ*_abs_ for **CBZ-F**. In contrast, the HOMO−1 and LUMO+1 orbital lobes were localized over the carbazole moiety. This result indicates that the HOMO−1 → LUMO+1 transition with a high transition energy is a local excitation (LE) transition. Similarly, in the case of **PIP-F** and **PIP-H**, the CT transition involved in the HOMO → LUMO transition had a low transition energy, and the LE transition involved in the HOMO → LUMO+2 transition had a high transition energy (Figure 4). In contrast, the **PTZ** analogs displayed an orthogonal molecular geometry between the tolane scaffold and phenothiazine moiety, which resulted in a large orbital separation between the HOMO and LUMO. Owing to the HOMO-LUMO orbital separation, the HOMO → LUMO transition is now a forbidden transition, and only one transition is allowed; HOMO−2 → LUMO in **PTZ-F** and HOMO−1 → LUMO in **PTZ-H** can be considered as LE transitions (Figure 4).

Subsequently, the PL behavior of the CH_2_Cl_2_ solutions of D-π-A-type tolanes was investigated. When the solution of **CBZ-F** in CH_2_Cl_2_ was irradiated with light (*λ*_abs_ in the long-wavelength absorption region), green PL with a single band at the maximum PL wavelength (*λ*_PL_) of approximately 542 nm was observed with a high PL efficiency (*Φ*_PL_ = 0.65) (Figure 3d). Although **CBZ-H** was found to emit intense blue PL, its PL behavior (*λ*_PL_ = 447 nm, *Φ*_PL_ = 1.0) was remarkably different from that of fluorinated **CBZ-F**. The PL bands for **CBZ-H** and **CBZ-F** can be attributed to the fluorescence involving the intramolecular charge transfer (ICT) from the donor to acceptor for the HOMO-LUMO transition, which can be reasonably explained by the solvent effect of absorption and PL behavior (Figure S19) [31,32]. The PL lifetimes (*τ*) of the tolanes were monitored at their corresponding *λ*_PL_. The *τ* was 5.38 ns for **CBZ-F** and 3.51 ns for **CBZ-H** (Figure S18); considering the nanosecond order of *τ*, the PL of the **CBZ** analogues can be considered as fluorescence. The radiation rate constant (*k*_r_) and non-radiation rate constant (*k*_nr_) were determined from the *Φ*_PL_ and *τ* values. The *k*_nr_ value of **CBZ-F** was larger than that of **CBZ-H**. Owing to the presence of fluorine atoms, the HOMO-LUMO energy gap (ΔE) in **CBZ-F** was lower than that in **CBZ-H**. This likely facilitates the non-radiative process, resulting in low *Φ*_PL_ based on the energy-gap law.

When the PL behavior of the **PIP** and **PTZ** analogs was measured in CH_2_Cl_2_ solution, the same PL tendency as that of the **CBZ** analogs was observed (Figure 3e,f). The PL band of the fluorinated analogs appeared in the longer-wavelength region than that of the corresponding non-fluorinated counterpart. Based on the Lippert–Mataga relationship, this PL behavior of the tolane analogs was probably caused by the ICT character resulting from the large electron-density distribution. **PIP-F** exhibited a long-wavelength PL because the excited state was stabilized owing to the large ICT characteristics. In contrast, **PTZ-F** resulted in a short-wavelength PL. **PIP** and **PTZ** analogs exhibited a significantly reduced *Φ*_PL_ (less than 0.26) with a large difference from that of the **CBZ** analog. This may have occurred for the **PIP** analogs because the strong amine-based donor therein rotated in the excited state to form a stable charge-separation state, called the twisted intramolecular charge transfer (TICT) state, resulting in a weak PL intensity and a rapid decay in PL (*τ* = 1.18 ns for **PIP-F**) due to non-radiative deactivation through dramatic structural relaxation of the donor moiety [33]. In the case of **PTZ** analogs, the orthogonal molecular geometry significantly reduces the electron-donating character of phenothiazine to the tolane moiety, causing fast PL decay (*τ* = 2.38 ns) on account of structural relaxation from the emissive ππ* excited state to the non-emissive πσ* excited state, resulting in low PL due to non-radiative deactivation [34,35].

### 2.3. Photophysical Properties in Crystalline State

**Figure 5:**
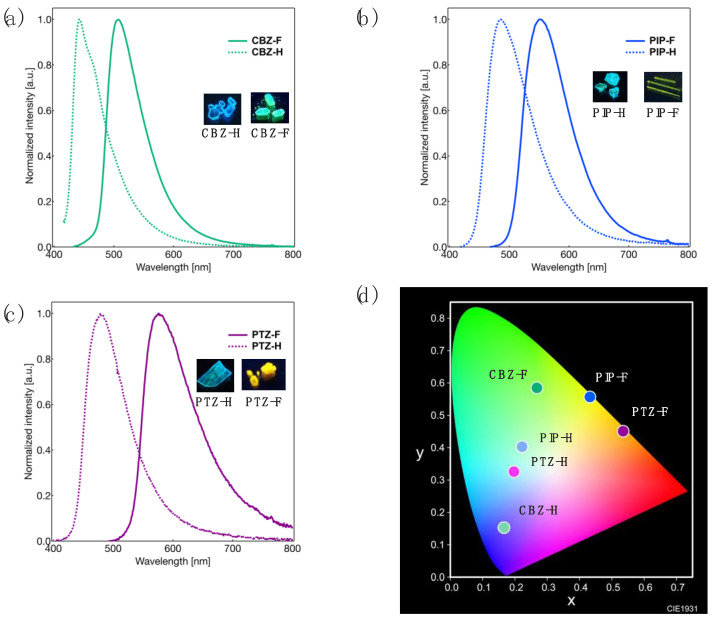
PL spectra of (**a**) **CBZ-F** and **CBZ-H**, (**b**) **PIP-F** and **PIP-H**, and (**c**) **PTZ-F** and **PTZ-H** in crystalline states. Excitation wavelength (*λ*_ex_): 300 nm (**CBZ-F**, **CBZ-H**, **PIP-H**, **PTZ-F**), 310 nm (**PIP-F**, **PTZ-H**). (**d**) CIE color diagram of all derivatives.

Next, we focused on the photophysical behavior of the synthesized D-π-A-type tolanes in their crystalline states. Figure 5 shows the measured PL spectra and the CIE color diagram, and the photophysical data obtained are summarized in Table 3.

Crystalline **CBZ-F** was found to exhibit relatively intense PL (*Φ*_PL_ = 0.42) with a single PL band at approximately 507 nm of *λ*_PL_ (Figure 4a). In contrast, **CBZ-H** emitted PL (*Φ*_PL_ = 0.48) with a *λ*_PL_ at approximately 442 nm in its crystalline state. Although **PIP-F** and **PTZ-F** exhibited a weak PL in solution, their crystalline samples were found to exhibit PL in the range of 0.16–0.18 *Φ*_PL_ with an *λ*_PL_ at approximately 551 nm for **PIP-F** and 575 nm for **PTZ-F** (Figure 4b,c). However, no significant improvement was observed in the PL behavior of their non-fluorinated counterparts. **PIP-H** showed similar PL in the crystalline (*λ*_PL_ = 485 nm, *Φ*_PL_ = 0.30) and solution states (*λ*_PL_ = 496 nm, *Φ*_PL_ = 0.26), and **PTZ-H** displayed a weak PL both in the crystalline (*λ*_PL_ = 478 nm, *Φ*_PL_ = 0.06) and solution states (*λ*_PL_ = 447 nm, *Φ*_PL_ = 0.02). Considering the Comission Internationale de l’Eclailage (CIE) color diagram, the PL color of **CBZ-F**, **PIP-F**, and **PTZ-F** was observed to be green, yellow, and orange, respectively, although the corresponding hydrogenated counterparts, viz., **CBZ-H**, **PIP-H**, and **PTZ-H**, were in a range of the blue to yellow region (Figure 5d).

**Figure 6:**
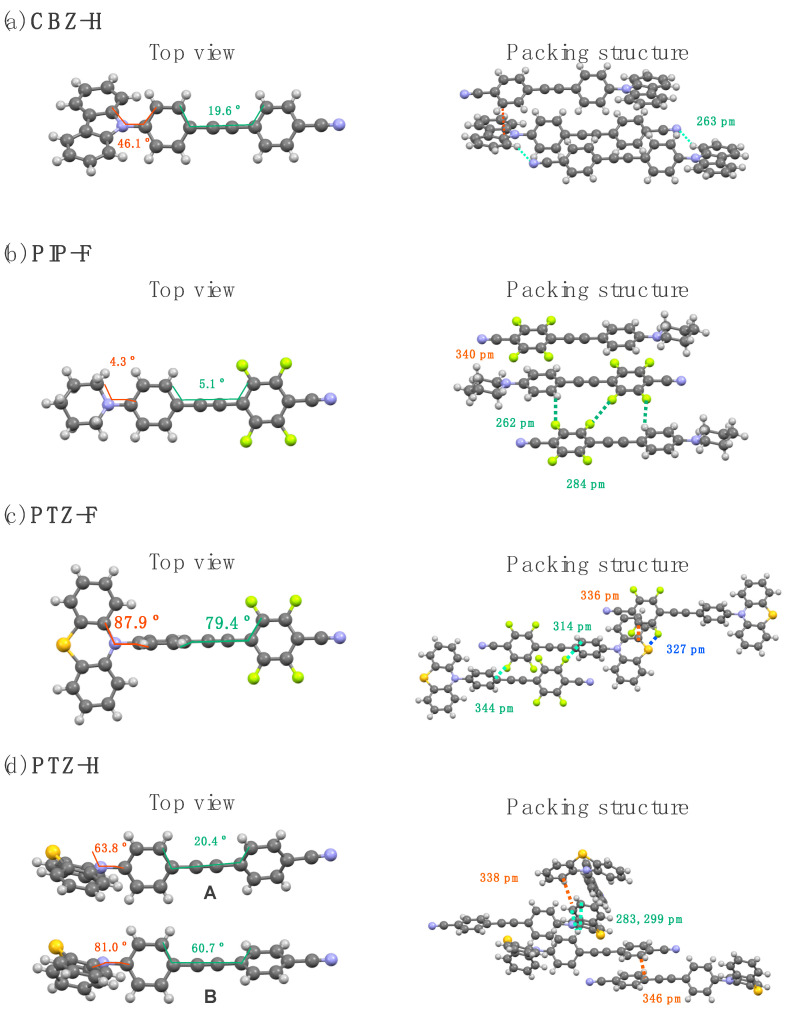
Crystal structure from top view, and the packing structures with interatomic short-contacts for (**a**) **CBZ-H**, (**b**) **PIP-F**, (**c**) **PTZ-F**, and (**d**) **PTZ-H**.

To understand the PL characteristics of the synthesized tolanes in the crystalline state, their molecular aggregated structures were investigated. After several recrystallization processes, the single crystals of **CBZ-H**, **PIP-F**, **PTZ-F**, and **PTZ-H** were used for X-ray crystallography. Figure 6 shows the crystal structures from the top view and the packing structures with an interatomic short contact for **CBZ- H**, **PIP-F**, **PTZ-F**, and **PTZ-H**.

**CBZ-H**, which contained the carbazole unit, crystallized in the monoclinic *P*12_1_/c space group, and four molecular units were contained in a unit cell. The tolane scaffold of **CBZ-H** has a planar structure, while the carbazole unit is twisted by 46.1° with respect to the tolane plane (Figure 6a). In the packing structures, several intermolecular π/π stacking interactions (π···π: 329 pm) between the electron-deficient aromatic ring and electron-rich carbazole unit and hydrogen bonds (N···H: 263 pm) between the hydrogen atom in the carbazolyl group and the nitrogen atom in the cyano group were observed. The interatomic distances for these interactions are less than the sum of their van der Waals radii (C: 170 pm, H: 120 pm, and N: 155 pm) [36].

**PIP-F** with a piperidine substituent crystallizes in the triclinic *P*–1 space group, and the unit cell contains two molecular units. The fluorinated tolane scaffold exhibits an almost planar structure with a 5.1° dihedral angle composed of two aromatic rings (Figure 6b). The dihedral angle formed by the piperidine ring and tolane plane was only 4.3°, which was found to be a flat conformation of the entire molecule. In the packing structures, as shown in Figure 5b, two fluorinated tolanes were packed in an antiparallel arrangement with π/π stacking interactions (π···π: 340 pm). In addition, the H···F hydrogen bonds between the fluorine and hydrogen atoms and the F/F interaction between fluorine atoms were observed (H···F: 262 pm, F···F: 284 pm) in the packing structures. The observed interatomic distances are also shorter than the sum of their van der Waals radii (F: 147 pm) [36].

**PTZ-F** with a phenothiazine unit belongs to the monoclinic *C*12/*c*1 space group and contains six molecules in a unit cell. With a large difference from the above crystal structures, that is, **CBZ-H** and **PIP-F**, two aromatic rings in the tolane scaffold were twisted at 79.4° (Figure 6c). Additionally, the phenothiazine unit was twisted at 87.9° with respect to the aromatic ring in the tolane scaffold. As shown in Figure 5c, because of the twisted tolane scaffold, the tolane molecules were tightly packed with a short interfacial distance (C···C: 336 pm) between the electron-deficient fluorinated aromatic ring and electron-rich phenothiazine unit. Additional intermolecular interactions involve S···F interactions (short contact: 327 pm) between sulfur in the phenothiazine unit and fluorine atoms and π···F interactions (short contact: 344 pm) between the phenothiazine unit attached to the electron-rich aromatic ring and fluorine atoms (S: 180 pm) [36].

**PTZ-H** forms crystal structures in the triclinic *P*–1 space group, and the unit cell contains four molecular units. In contrast to **CBZ- H**, **PIP-F**, and **PTZ-F**, **PTZ-H** exhibited two conformational structures: one of the two aromatic rings in the tolane scaffold of conformer **A** possessed a twisted structure of 20.4°, whereas conformer **B** was twisted at 60.7°. In both conformers, the phenothiazine unit was twisted at 63.8° for **A** and 81.0° for **B** with respect to the aromatic ring in the tolane scaffold (Figure 6d). As shown in Figure 5d, the more twisted **B** intermolecularly formed loose π/π stacking with a short contact of 346 pm. Additional π/π stacking (C···C 338 pm) between the two phenothiazine units is also observed in the packing structures. Moreover, CH···π interactions (interatomic distance: 283 and 299 pm) between the carbon and hydrogen atoms between the two phenothiazine units were confirmed intermolecularly.

From the above crystal structure, the following points can be reasonably explained:

(i) The *Φ*_PL_ of **CBZ-H** in the crystalline state is lower than that in the solution state because of the promotion of non-radiative deactivation through intermolecular energy transfer via tight π/π stacking.
(ii) The increase in the *Φ*_PL_ of **PIP-F** and **PTZ-F** in the crystalline state compared to that in the solution state is due to the effective suppression of non-radiative deactivation and the formation of rigid molecular aggregated structures. This is supported by the significant reduction in the nonradiative rate constant (*k*_nr_) in the crystalline state, rather than in the solution state.
(iii) Comparing PL decay among the three fluorinated tolanes, viz., **CBZ-F**, **PIP-F**, and **PTZ-F**, however, irregular PL decay profiles were observed depending on the donor moiety. This can be attributed to the significant alteration of molecular aggregated structures in the crystalline state. Thus, the retardation of PL in case of **CBZ-F** and **PTZ-F** crystals is due to the dimer-like molecular packing induced by strong CT interactions between the electron-donating aromatic ring-substituted amine-donor moieties and the electron-withdrawing fluorinated aromatic rings. In contrast, **PIP-F** crystals demonstrated rapid PL decay due to structural relaxation of the donor moiety, which is similar to solution-state PL decay.
(iv) The red-shift of the PL band of **PTZ-F** in the crystalline state compared to that in the solution state is due to the formation of dimer-like molecular packing structures through tight π/π stacking caused by the orthogonal molecular structure characteristic of **PTZ-F** [15]. This is supported by the significantly delayed *τ* in **PTZ-F** compared with that in **CBZ-F** and **PIP-F** (Figure S19).

### 2.4. Aggregation-Induced Emission Behavior

**Figure 7:**
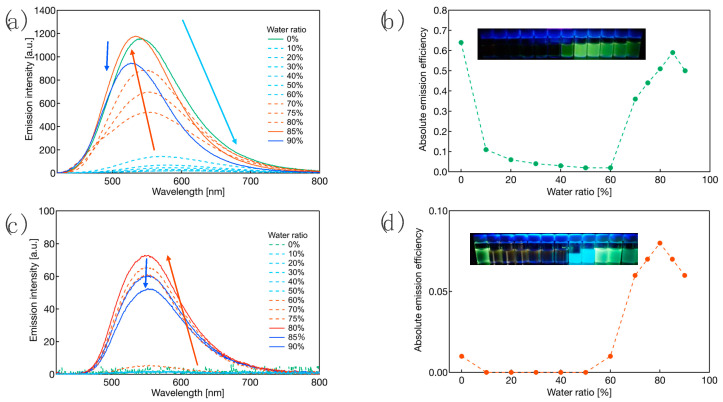
(**a**) PL spectra in THF/H_2_O mixed solution; (**b**) the relationship between *Φ*_PL_ and water fraction of **CBZ-F**; (**c**) PL spectra in THF/H_2_O mixed solution; (**d**) the relationship between *Φ*_PL_ and water fraction of **PIP-F**. Insets: the photographs of the states of light emission irradiated by a UV lamp (*λ*_ex_: 365 nm).

The phenomenon in which the *Φ*_PL_ of **CBZ-F** decreases in polar solvents, as shown in Table S9, can be applied to the aggregation-induced emission (AIE) phenomenon of **CBZ-F**. In addition, the *Φ*_PL_ of **PIP-F** is enhanced in the crystalline state and not in its solution state. Therefore, we assumed that **CBZ-F** and **PIP-F** show aggregation-induced emission (AIE) behavior as a selected example. Therefore, we evaluated the AIE behavior of **CBZ-F** and **PIP-F** by dissolving their crystalline samples in THF. Subsequently, their PL intensity and *Φ*_PL_ were assessed in a mixed solution with varying proportions of THF and water. Figure 7 shows the AIE behavior of **CBZ-F** and **PIP-F**.

**CBZ-F** showed an intense PL with a high *Φ*_PL_ (0.64) in the THF solution. When the addition ratio of the water fraction was gradually increased, the PL intensity of **CBZ-F** gradually decreased with decreasing *Φ*_PL_ (Figure 7a,b), which was due to the significant acceleration of the non-radiative deactivation process induced by the increasing solvent polarity. When 60% water was added, the PL intensity was reduced to approximately one fifty-third of that of 100% THF (Figure 7a). Molecular aggregation was initiated by the addition of water (Figure S22), and a gradual increase in the PL intensity was observed, resulting in a yellow PL. When the water fraction reached 85%, the PL intensity and *Φ*_PL_ recovered to almost the same value (0.59) as that of 100% THF (Figure 7b). The addition of more than 85% water reduced the PL efficiency owing to the polar effect of the solvent and the quenching effect caused by aggregation. In contrast to **CBZ-F**, **PIP-F** displayed very little PL in the water fraction range of 0–60 %. However, when the water ratio reached 70%, the PL intensity dramatically increased after forming molecular aggregates (Figure S22), and the maximum PL intensity with the highest *Φ*_PL_ (0.08) was achieved when the water fraction reached 80% (Figure 7c,d). Accordingly, both **CBZ-F** and **PIP-F** showed AIE characteristics, which could pave the way for the development of promising solid-state yellow-luminescent materials. Analyzing the PL behavior in THF and polyethylene glycol (PEG) mixed solvent, increasing the PEG fraction, the PL intensity and *Φ*_PL_ monotonically decreased and a slight long-wavelength shift was observed for **CBZ-F** (Figure S21a), which is due to the solute–solvent interaction induced by increasing solvent polarity. On the other hand, the PL intensity and *Φ*_PL_ of **PIP-F** decreased monotonically below 50% PEG fraction, but increased gradually when the PEG fraction exceeded 60% (Figure S21b), which is due to restriction of intramolecular motion (RIM) caused by increasing solvent viscosity. Judging from the above results, the AIE phenomenon of **CBZ-F** is considered to be mainly caused by the intermolecular H···F hydrogen bonds, not the dynamic structural relaxation effect, whereas the AIE phenomenon of **PIP-F** is considered to be caused by the RIM mechanism, in which the rotational motion of the piperidyl group is suppressed.

## 3. Materials and Methods

### 3.1. General Method

All reactions were performed using dried glassware and magnetic stirrer bars. All chemicals were of reagent grade and purified in the usual manner prior to use. Wakogel^®^ 60 N, 38–100 μm), and TLC analysis was performed on silica gel TLC plates (Merck, Silica gel 60F_254_). ^1^H and ^13^C NMR spectra were obtained using a Bruker AVANCE III 400 NMR spectrometer (^1^H: 400 MHz and ^13^C: 100 MHz) in chloroform-*d* (CDCl_3_), and chemical shifts were reported in parts per million (ppm) using the residual proton in the NMR solvent. ^19^F NMR (376 MHz) spectra were obtained using a Bruker AVANCE III 400 NMR spectrometer in CDCl_3_, and CFCl_3_ (*δ*_F_ = 0.0 ppm) or hexafluorobenzene (*δ*_F_ = −163 ppm) were used as internal standards. Infrared spectra (IR) spectra were recorded using the KBr method with a JASCO FT/IR-4100 type A spectrometer; all spectra were reported in wavenumber (cm^−1^). High-resolution mass spectra (HRMS) were recorded on a JEOL JMS-700MS spectrometer using the fast atom bombardment (FAB) method.

### 3.2. Synthesis

#### 3.2.1. Typical Procedure for the Nucleophilic Aromatic Substitution Reaction of Lithium 4-Carbazol-9-ylphenylacetylide with Pentafluorobenzonitrile

In a two-necked round-bottomed flask, 4-carbazol-9-ylphenylacetylene (1.1 g, 4.2 mmol) was added to THF (40 mL), and the mixture was cooled to 0 °C. *n*-butyllithium (1.6 mol L^−1^ hexane solution, 3.9 mL, 6.2 mmol) was added dropwise to this mixture, and the resulting solution was continuously stirred at 0 °C for 0.5 h. Pentafluorobenzonitrile (0.80 mL, 6.2 mmol) was then added dropwise to the solution. After the addition was complete, the reaction mixture was warmed to 25 °C and stirred for an additional 18 h. The reaction mixture was then poured into a saturated aqueous NH_4_Cl solution, the crude product was extracted with ethyl acetate (EtOAc) three times, and the combined organic layer was washed with brine. The organic layer was dried over anhydrous sodium sulfate (Na_2_SO_4_), which was then separated by filtration. The filtrate was evaporated in vacuo and purified by silica-gel column chromatography (eluent: hexane/EtOAc = 10/1), followed by recrystallization from a mixed solvent system (hexane/CHCl_3_ = 1/1), which afforded the corresponding fluorinated tolane, **CBZ-F**, in 15% isolated yield (0.27 g, 0.61 mmol) as green crystals.

##### 4-(2-(4-Carbazol-9-ylphenyl)ethynyl)-2,3,5,6-Tetrafluorobenzonitrile (**CBZ-F**)

Yield: 15% (green crystal); M.p.: 210.9–213.1 °C; ^1^H NMR (CDCl_3_): *δ* 7.33 (t, *J* = 7.9 Hz, 2H), 7.49–7.42 (m, 4H), 7.68 (d, *J* = 8.6 Hz, 2H), 7.86 (d, *J* = 8.6 Hz, 2H), 8.15 (d, *J* = 7.7 Hz, 2H); ^13^C NMR (CDCl_3_): *δ* 74.5, 93.8, 106.1, 107.5, 109.8, 111.1, 119.4, 120.1, 120.7, 123.9, 126.4, 127.0, 134.0, 140.0, 140.3, 146.7 (dm, *J* = 265.6 Hz), 147.2 (dm, *J* = 266.8 Hz); ^19^F NMR (CDCl_3_, CFCl_3_); *δ* −134.6 (m, 2F), −133.9 (m, 2F); IR (KBr) *ν* 3056, 2222, 1647, 1602, 1559, 1520, 1489, 1450, 1356, 1335, 1242, 1188, 1077, 984 cm^−1^; HRMS: (FAB+) *m*/*z* [M]^+^ calcd for C_27_H_12_F_4_N_2_: 440.0937, Found: 440.0934.

##### 4-(2-(4-Piperidylphenyl)ethynyl)-2,3,5,6-Tetrafluorobenzonitrile (**PIP-F**)

Yield: 45% (yellow crystal); M.p.: 182.4–184.1 °C; ^1^H NMR (CDCl_3_): *δ* 1.55–1.66 (m, 6H), 3.32 (t, *J* = 4.8 Hz, 4H), 6.86 (d, *J* = 8.8 Hz, 2H), 7.46 (d, *J* = 8.8 Hz, 2H); ^13^C NMR (CDCl_3_): *δ* 24.4, 25.5, 48.9, 73.2, 92.0, 107.9, 108.6, 109.6, 112.3, 114.4, 133.8, 146.1 (dm, *J* = 251.2 Hz), 147.1 (dm, *J* = 258.4 Hz), 152.7; ^19^F NMR (CDCl_3_): *δ* −136.05 (m, 2F), −134.90 (m, 2F); IR (KBr): *δ* 3286, 3044, 2947, 2845, 2567, 2480, 2242, 2211, 1896, 1597, 1542, 5121, 1492, 1458, 1426, 1335, 1243, 1168, 1001, 930 cm^−1^; HRMS: (FAB+) *m*/*z* [M]^+^ calcd for C_27_H_14_F_4_N_2_: 358.1093; found: 358.1099.

##### 4-(2-(4-Phenothiazin-10-ylphenyl)ethynyl)-2,3,5,6-Tetrafluorobenzonitrile (**PTZ-F**)

Yield: 18% (orange crystal); M.p.: 201.3–201.9 °C; ^1^H NMR (CDCl_3_): *δ* 6.84 (d, *J* = 8.0 Hz, 2H), 7.04 (t, *J* = 7.7 Hz, 2H), 7.12 (t, *J* = 7.7 Hz, 2H), 7.24 (d, *J* = 8.8 Hz, 2H), 7.27 (d, *J* = 8.8 Hz, 2H), 7.62 (d, *J* = 8.8 Hz, 2H); ^13^C NMR (CDCl_3_): *δ* 74.1, 93.3, 107.1, 116.4, 121.1, 121.9, 123.0, 124.2, 124.5, 124.8, 127.2, 127.9, 128.1, 134.2, 142.4, 146.52 (dm, *J* = 259.6 Hz), 145.8, 147.2 (dm, *J* = 259.3 Hz); ^19^F NMR (CDCl_3_, C_6_F_6_): *δ* −134.90 (m, 2F), −135.08 (m, 2F); IR (KBr): *δ* 3066, 3008, 2260, 2246, 2230, 2027, 1932, 1605, 1559, 1512, 1457, 1443, 1431, 1412, 1405, 1310, 1164, 1017 cm^−1^; HRMS: (FAB+) *m*/*z* [M]^+^ calcd for C_27_H_12_F_4_N_2_S: 472.0657; found: 472.0651.

#### 3.2.2. Typical Procedure for the Pd(0)-Catalyzed Sonogashira Cross-Couplilng Reaction of 4-Carbazol-9-ylphenylacetylene with 4-Bromobenzonitrile

In a two-necked round-bottomed flask were added 4-carbazol-9-ylphenylacetylene (1.3 g, 5.0 mmol), dichlorobis(triphenylphosphine)palladium(0) (0.18 g, 0.25 mmol), triphenylphosphine (0.066 g, 0.25 mmol), copper(I) iodide (0.095 g, 0.50 mmol), 4-bromobenzonitrile (1.1 g, 6.0 mmol), and triethylamine (15 mL). The resultant mixture was stirred at 80 °C for 20 h. The precipitate formed during the reaction was separated by atmospheric filtration, and the filtrate was poured into a saturated aqueous NH_4_Cl solution. The crude product was extracted three times with ethyl acetate (EtOAc), and the combined organic layer was washed once with brine. The organic layer was dried over anhydrous sodium sulfate (Na_2_SO_4_), which was separated by filtration. The filtrate was evaporated in vacuo and purified by silica-gel column chromatography (eluent: hexane/EtOAc = 20/1), followed by recrystallization from a mixed solvent system (hexane/CH_2_Cl_2_ = 1/1) to afford the corresponding fluorinated tolane, **CBZ-H**, in 38% isolated yield (0.71 g, 1.9 mmol) as colorless crystals.

##### 4-(2-(4-Carbazol-9-ylphenyl)ethynyl)benzonitrile (**CBZ-H**)

Yield: 38% (colorless crystal); M.p.: 221.1–222.0 °C; ^1^H NMR (CDCl_3_): *δ* 7.33 (t, *J* = 3.2 Hz, 2H), 7.41–7.47 (m, 4H), 7.63 (d, *J* = 8.8 Hz, 2H), 7.65–7.69 (m, 4H), 7.78 (d, *J* = 8.8 Hz, 2H), 8.16 (d, *J* = 8.8 Hz, 2H); ^13^C NMR (CDCl_3_): *δ* 88.7, 93.1, 109.8, 111.9, 118.6, 120.5, 120.6, 121.2, 123.8, 126.3, 127.0, 128.1, 132.3, 133.5, 135.6, 149.6; IR (KBr) *ν* 3067, 3042, 2537, 2230, 2215, 1911, 1541, 1479, 1405, 1366, 1271, 1150, 1003 cm^−1^; HRMS (FAB) *m*/*z* [M]^+^ calcd for (M^+^) C_27_H_16_N_2_: 368.1313, Found: 368.1321.

##### 4-(2-(4-Piperidylphenyl)ethynyl)-2,3,5,6-Tetrafluorobenzonitrile (**PIP-H**)

Yield: 8% (colorless crystal); M.p.: 188.0–190.6 °C; ^1^H NMR (CDCl_3_): *δ* 1.62–1.69 (m, 6H), 3.26 (t, *J* = 2.8 Hz, 4H), 6.86 (d, *J* = 8.8 Hz, 2H), 7.40 (d, *J* = 8.8 Hz, 2H), 7.55–7.60 (m, 4H); ^13^C NMR (CDCl_3_): *δ* 24.5, 25.6, 49.4, 86.6, 95.5, 110.7, 111.0, 115.1, 118.8, 129.3, 131.8, 132.1, 133.1, 152.1 ; IR (KBr): *ν* 3399, 3091, 3042, 2942, 2855, 2222, 2209, 2167, 1891, 1685, 1606, 1543, 1466, 1451, 1445, 1425, 1407, 1262, 1197, 1026 cm^−1^; HRMS: (FAB+) *m*/*z* [M]^+^ calcd for C_27_H_14_F_4_N_2_: 268.1470; found: 268.1473.

##### 4-(2-(4-Phenothiazin-10-ylphenyl)ethynyl)benzonitrile (**PTZ-H**)

Yield: 38% (white crystal); M.p.: 174.3–175.2 °C; ^1^H NMR (CDCl_3_): *δ* 6.53 (d, *J* = 8.0 Hz, 2H), 6.94 (t, *J* = 7.4 Hz, 2H), 7.00 (t, *J* = 8.4 Hz, 2H), 7.15 (d, *J* = 8.8 Hz, 2H), 7.32 (d, *J* = 8.4 Hz, 2H), 7.61–7.69 (m, 6H); ^13^C NMR (CDCl_3_): *δ* 88.6, 93.3, 111.7, 118.6, 118.9, 120.3, 123.7, 124.1, 127.1, 127.2, 127.5, 128.2, 132.2, 132.2, 134.0, 143.1, 143.3 ; IR (KBr): *ν* 3085, 3075, 3069, 3060, 3042, 3021, 3016, 3007, 2229, 2176, 1921, 1793, 1457, 1405, 1379, 1270, 1188, 1156, 1064 cm^−1^; HRMS: (FAB+) *m*/*z* [M]^+^ calcd for C_27_H_16_N_2_S: 400.1034; found: 400.1038.

### 3.3. Single Crystal X-ray Diffraction

Single-crystal X-ray diffraction spectra were recorded using an XtaLAB AFC11 diffractometer (Rigaku, Tokyo, Japan). The reflection data were integrated, scaled, and averaged using the CrysAlisPro program (ver. 1.171.39.43a; Rigaku Corporation, Akishima, Japan). Empirical absorption corrections were applied using the SCALE 3 ABSPACK scaling algorithm (CrysAlisPro). The structures were identified by a direct method (SHELXT-2018/2 [37]) and refined using the full matrix least-squares method (SHELXL-2018/3 [38]) visualized by Olex2 [39]. Crystallographic data were deposited in the Cambridge Crystallographic Data Centre (CCDC) database (CCDC 2151065 for **PIP-F**, 2151066 for **CBZ-H**, 2151067 for **PTZ-H**, and 2151068 for **PTZ-F**). These data can be obtained free of charge from the CCDC at www.ccdc.cam.ac.uk/data_request/cif (accessed on 4 September 2022).

### 3.4. Photophysical Properties

UV–vis absorption spectra were recorded using a JASCO V-750 absorption spectrometer (JASCO, Tokyo, Japan). The PL spectra of the solutions were measured using a FP-6600 fluorescence spectrometer (JASCO, Tokyo, Japan). Photoluminescence quantum yields were measured using a Quantaurus-QY C11347-01 instrument (Hamamatsu Photonics, Hamamatsu, Japan). The PL lifetime was measured using a Quantaurus-Tau fluorescence lifetime spectrometer (C11367-34, Hamamatsu Photonics, Japan).

### 3.5. Theoretical Calculation

All computations were performed using density functional theory (DFT) at the M06-2X hybrid functional [29] and 6-31 + G(d,p) (for all atoms) basis set with a conductor-like polarizable continuum model (CPCM) [30] for CH_2_Cl_2_ using the Gaussian 16 program package [28]. Theoretical vertical transitions were also calculated using a time-dependent DFT (TD-DFT) method at the same level of theory using the same solvation model.

## 4. Conclusions

We synthesized three types of fluorinated tolanes with amine-based donors and evaluated their photophysical behavior in detail. The carbazole-substituted fluorinated tolane showed intense yellow PL in its dilute solution state; however, piperidine or a phenothizine-substituted tolanes exhibited a significant decrease in PL efficiency (*Φ*_PL_), which was caused by the twisted intramolecular charge transfer (TICT) process or an immediate internal conversion of the tolane scaffold from ππ* to dark πσ* excited states. Interestingly, fluorinated tolanes exhibited PL in the crystalline state. Moreover, fluorinated tolanes with piperidine or phenothiazine substituents showing weak PL properties in solution were found to show improved PL efficiencies in their crystalline states, leading to yellow-to-orange PL. Crystal structure analysis shows that the tight packing structures formed in the crystal are attributable to the significant suppression of non-radiative deactivation, leading to a dramatic improvement in *Φ*_PL_. Furthermore, the *Φ*_PL_ of the non-fluorinated tolanes did not increase in their crystalline state; this suggests that the introduction of fluorine atoms into the tolane scaffold strongly influences the formation of aggregated structures via H···F hydrogen bonds. In addition, we confirmed that the photophysical behavior of fluorinated tolanes showed aggregation-induced emission behavior. These findings provide guidelines for the molecular design of new solid-state luminescent materials, and we believe that functional materials based on this molecular design approach will emerge in the near future.

## Figures and Tables

**Table 1:** Photophysical data of the D-π-A-type tolanes obtained in CH_2_Cl_2_ solution and their calculated data.

	*λ*_abs_ [nm] ^1^	HOMO ^2^	LUMO ^2^	ΔE^H−L^	*λ*_PL_ [nm] ^1,3^	*τ*_AVE_	*τ*_1_	*τ*_2_	*k*_r_^5^	*k*_nr_^6^
	(*ε* × 10^−3^ L mol^−1^ cm^−1^)	[eV]	[eV]	[eV]	(*Φ*_PL_) ^4^	[ns]	[ns]	[ns]	[10^7^ s^−1^]	[10^7^ s^−1^]
**CBZ-F**	238 (52), 291 (39), 375 (20)	–7.03	–2.09	4.94	542 (0.65)	5.38	–	–	12.1	6.5
**CBZ-H**	238 (52), 292 (39), 342 (31)	–6.97	–1.58	5.39	447 (1.0)	3.51	–	–	28.5	0.0
**PIP-F**	277 (30), 408 (44)	–6.94	–1.93	5.01	603 (0.02)	1.18	1.02	4.40	1.7	83.1
**PIP-H**	266 (27), 363 (35)	–6.60	–1.34	5.26	496 (0.26)	2.50	–	–	10.4	29.6
**PTZ-F**	258 (45), 307 (26), 326 (23), 393 (21)	–6.75	–2.12	4.63	501 (0.01)	2.38	<1.0	3.39	0.42	41.6
**PTZ-H**	258 (49), 299 (32), 317 (33), 356 (13)	–6.72	–1.60	5.12	447 (0.02)	1.03	<1.0	5.14	1.9	95.2

^1^ Concentration: 1.0 × 10^−5^ mol L^−1^. ^2^ Calculated with density functional theory using the M06-2X/6-31 + G(d) level of theory and a conductor-like polarizable continuum model for CH_2_Cl_2_. ^3^ Excited at the longest absorption maximum wavelength. ^4^ Determined using an integrating sphere. ^5^ Radiation rate constant: *k_r_* = *Φ*_PL_/*τ*_AVE_. ^6^ Non-radiation rate constant: *k*_nr_ = (1 − *Φ*_PL_)/*τ*_AVE_.

**Table 2:** Theoretical values of vertical transition behavior calculated using TD-DFT ^1^.

Molecule	Theoretical Transition	Theoretical Transition Energy [eV]	Theoretical *λ*_abs_ [nm]	Oscillator Strength
**CBZ-F**	HOMO → LUMO	3.4871	355.55	1.3880
	HOMO–1 → LUMO+1	4.7119	263.13	0.4074
**CBZ-H**	HOMO → LUMO	3.7348	331.97	1.6078
	HOMO−1 → LUMO+1	4.7075	263.37	0.5154
**PIP-F**	HOMO → LUMO	3.3143	374.09	1.6677
	HOMO → LUMO+2	5.1695	239.84	0.2601
**PIP-H**	HOMO → LUMO	3.5015	354.09	1.7485
	HOMO → LUMO+3	4.4592	278.04	0.0599
**PTZ-F**	HOMO → LUMO	3.4666	357.65	0.0000
	HOMO−2 → LUMO	3.9363	314.98	1.7773
**PTZ-H**	HOMO → LUMO	3.7800	328.00	0.0003
	HOMO−1 → LUMO	4.0564	305.65	1.8205

^1^ Calculated with density functional theory using the M06-2X/6-31 + G(d) level of theory and a conductor-like polarizable continuum model for CH_2_Cl_2_.

**Table 3:** Photophysical data of D-π-A-type tolane with an amine-based donor in crystal.

	*λ*_PL_ [nm] ^1^	*τ*_AVE_	*τ*_1_	*τ*_2_	*k*_r_^3^	*k*_nr_ ^4^
	(*Φ*_PL_) ^2^	[ns]	[ns]	[ns]	[10^7^ s^−1^]	[10^7^ s^−1^]
**CBZ-F**	507 (0.42)	52.8	19.4	62.1	0.8	1.1
**CBZ-H**	442 (0.48)	5.32	2.67	5.27	9.0	9.8
**PIP-F**	551 (0.18)	8.82	7.41	13.1	2.0	9.3
**PIP-H**	485 (0.30)	3.18	1.95	5.75	9.4	22.0
**PTZ-F**	575 (0.16)	93.1	32.0	276	0.2	0.9
**PTZ-H**	478 (0.06)	2.31	–	–	2.6	40.7

^1^ Excited at 300 nm for **CBZ-F**, **CBZ-H**, **PIP-H**, **PTZ-F**, and at 310 nm for **PIP-F** and **PTZ-H**. ^2^ Determined using an integrating sphere. ^3^ Radiation rate constant: *k_r_* = *Φ*_PL_/*τ*_AVE_. ^4^ Non-radiation rate constant *k*_nr_ = (1 − *Φ*_PL_)/*τ*_AVE_.

## Data Availability

Not applicable.

## Supplementary Materials

The following are available online at https://www.mdpi.com/article/10.3390/molecules27185782/s1, Figure S1: Synthetic method of the target compounds; Figures S2–S15: NMR spectra of new compounds; Figure S16: UV–vis and PL spectra in CH_2_Cl_2_ solution; Figure S17: Excitation and PL spectra in crystalline state; Figures S18 and S19: PL decay curve of the target compounds; Figure S20: UV-vis and PL spectra of **CBZ-F**, **CBZ-H**, **PIP-F**, and **PTZ-F** in various solvents; Figure S21: PL spectra of **CBZ-F** and **PIP-F** in THF/PEG mixed solution; Figure S22: DLS profiles of **CBZ-F** and **PIP-F** in THF/H_2_O mixed solution; Table S1: Crystallographic data of **CBZ-H**, **PIP-F**, **PTZ-F**, and **PTZ-H**; Table S2: dipole moment of all compounds calculated by TD-DFT calculation; Tables S3–S8: Cartesian coordinates for optimized geometries; Tables S9–S12: Photophysical data of **CBZ-F**, **CBZ-H**, **PIP-F**, and **PTZ-F** in various solvent; Tables S13 and S14: Photophysical data of **CBZ-F** and **PIP-F** in THF/H_2_O mixed solution; Tables S15 and S16: Photophysical data of **CBZ-F** and **PIP-F** in THF/PEG mixed solution.

## Sample Availability

Samples of the compounds are available from the authors.
