# Supplementary material for: Development of Yellow-to-Orange Photoluminescence Molecules Based on Alterations in the Donor Units of Fluorinated Tolanes
Source: Molecules. 2022 Sep 7;27(18):5782. doi: 10.3390/molecules27185782 (PMC9504459; doi:10.3390/molecules27185782)
Supplement: Supplementary file 1 [file molecules-27-05782-s001.zip › molecules-1897730-supplementary.pdf]

Electronic Supplementary Information

Development of yellow-to-orange photoluminescence molecules based on alterations in the donor units of fluorinated tolanes

Shigeyuki Yamada [1,*], Kazuki Kobayashi [1], and Tsutomu Konno [1]

[1]Faculty of Molecular Chemistry and Engineering, Kyoto Institute of Technology, Matsugasaki, Sakyo-ku, Kyoto 606-8585, Japan.

*Correspondence: syamada@kit.ac.jp (S.Y.); Tel.: +81-75-724-7517

Table of Contents

1. Synthesis ●●●●●●●●●●● S-2
2. NMR Spectra ●●●●●●●●●●● S-3
3. X-ray crystallographic analysis ●●●●●●●●●●● S-12
4. DFT Calculation ●●●●●●●●●●● S-12
    Cartesian coordinate ●●●●●●●●●●● S-13
5. Photophysical Characteristics ●●●●●●●●●●● S-19
6. DLS measurement ●●●●●●●●●●● S-25

1. Synthesis

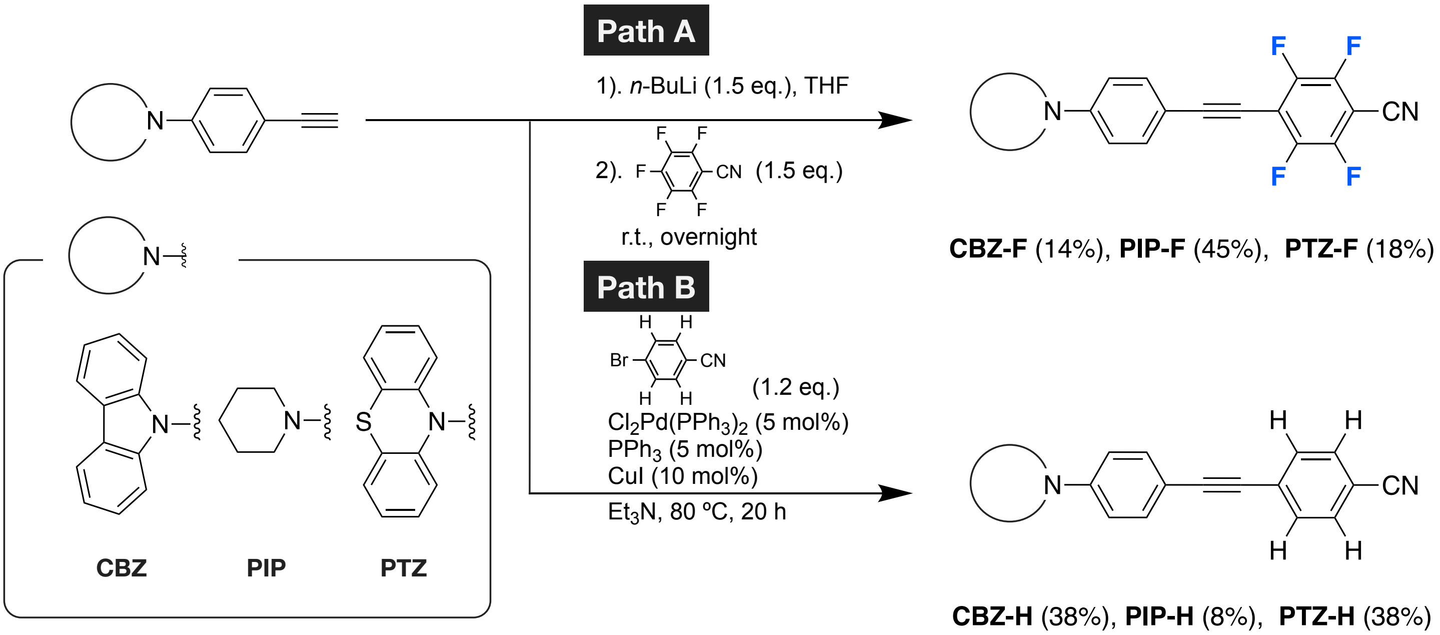

Typical synthesis procedure for Fluorinated tolanes: Path A

In a two-necked round-bottomed flask, 4-aminophenylacetylene was added to THF, and the mixture was cooled to 0 °C. *n*-butyllithium (1.6 mol L^{-1} hexane solution) was added dropwise to this mixture, and the resulting solution was continuously stirred at 0 °C for 0.5 h. Pentafluorobenzonitrile was then added dropwise to the solution. After the addition was complete, the reaction mixture was warmed to 25 °C and stirred for overnight. The reaction mixture was then poured into a saturated aqueous NH_4Cl solution, the crude product was extracted with ethyl acetate (EtOAc) three times, and the combined organic layer was washed with brine. The organic layer was dried over anhydrous sodium sulfate (Na_2SO_4), which was then separated by filtration. The filtrate was evaporated in vacuo and purified by silica-gel column chromatography (eluent: hexane/EtOAc = 10/1), followed by recrystallization from a mixed solvent system (hexane/$CHCl_3$ = 1/1), which afforded the corresponding fluorinated tolanes, in 14-45% isolated yield.

Typical synthesis procedure for non-Fluorinated tolanes: Path B

In a two-necked round-bottomed flask were added 4-aminophenylacetylene, dichlorobis(triphenylphosphine)palladium(0), triphenylphosphine, copper(I) iodide, 4-bromobenzonitrile, and triethylamine. The resultant mixture was stirred at 80 °C for 20 h. The precipitate formed during the reaction was separated by atmospheric filtration, and the filtrate was poured into a saturated aqueous NH_4Cl solution. The crude product was extracted three times with ethyl acetate (EtOAc), and the combined organic layer was washed once with brine. The organic layer was dried over anhydrous sodium sulfate (Na_2SO_4), which was separated by filtration. The filtrate was evaporated in vacuo and purified by silica-gel column chromatography (eluent: hexane/EtOAc = 20/1), followed by recrystallization from a mixed solvent system (hexane/CH_2Cl_2 = 1/1) to afford the corresponding non-fluorinated tolane, in 8-38% isolated yield

2. NMR Spectra

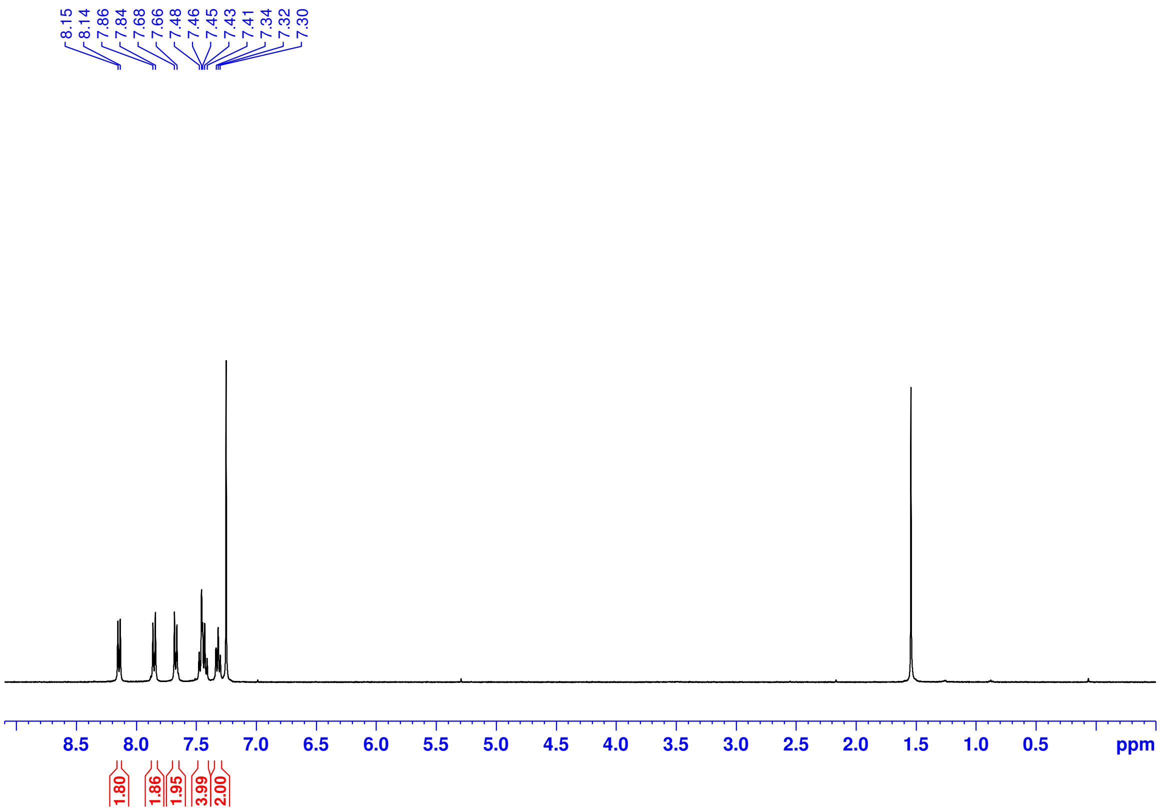

Figure S1. ^{1}H NMR spectrum of **CBZ-F** (400 MHz, $CDCl_3$).

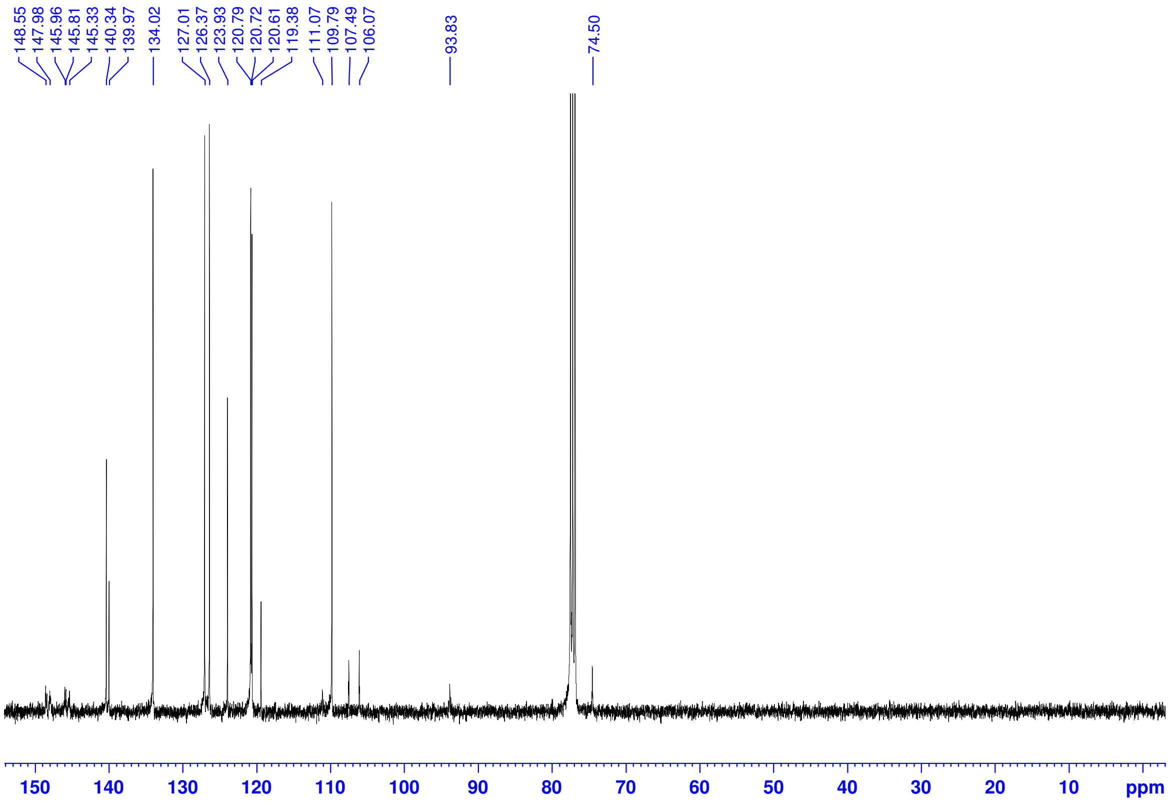

Figure S2. ^{13}C NMR spectrum of **CBZ-F** (100 MHz, $CDCl_3$).

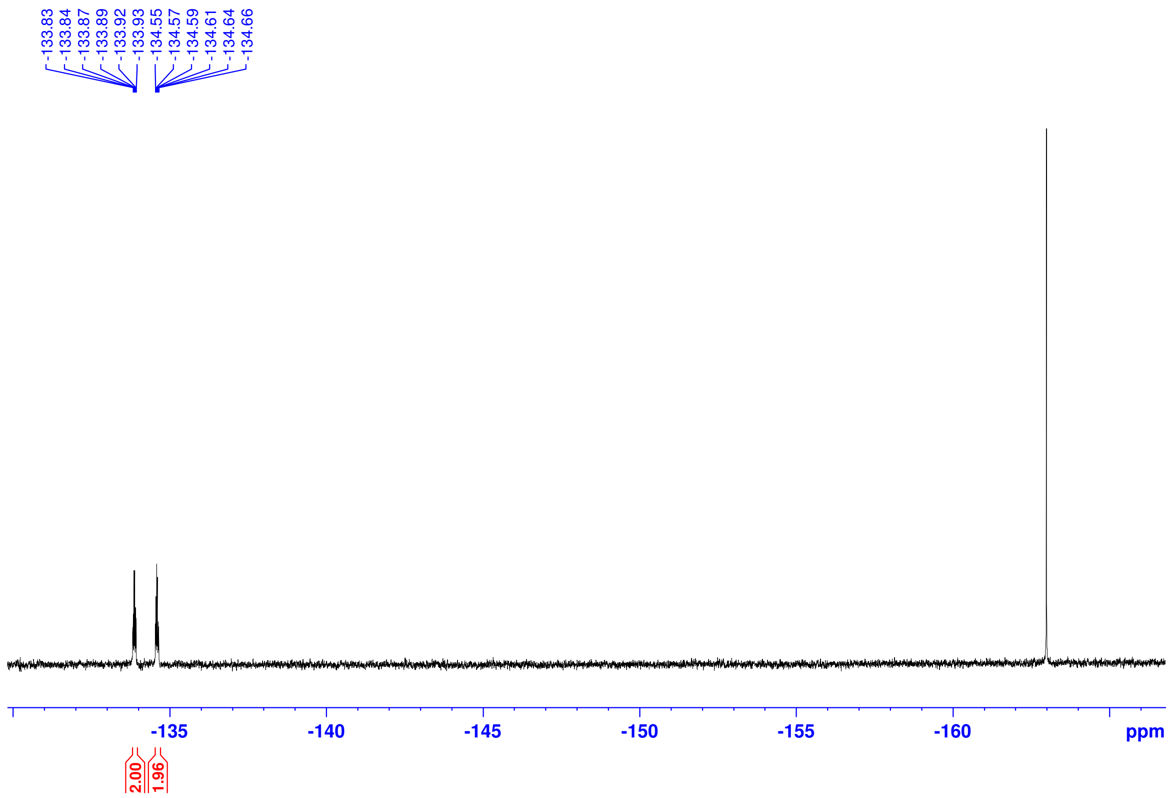

Figure S3. ^{19}F NMR spectrum of C**BZ-F** (376 MHz, $CDCl_3$, hexafluorobenzene δ_F = –163 ppm).

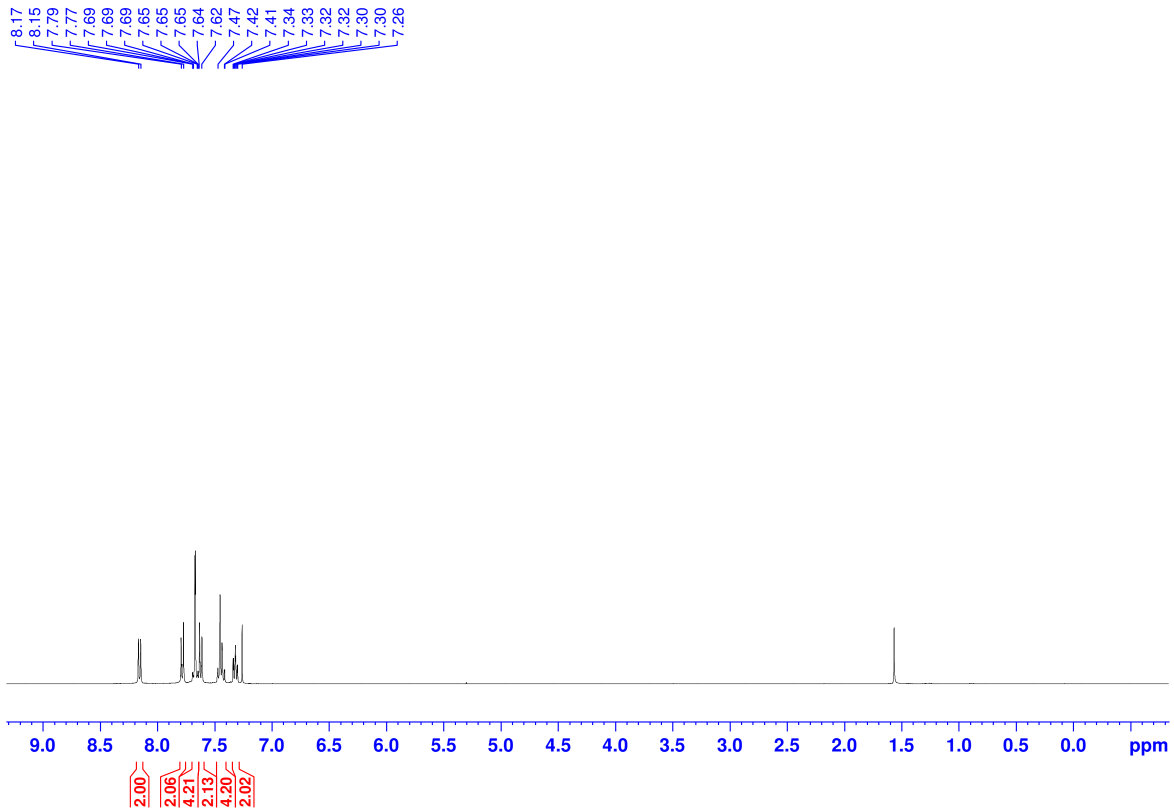

Figure S4. ^{1}H NMR spectrum of **CBZ-H** (400 MHz, $CDCl_3$).

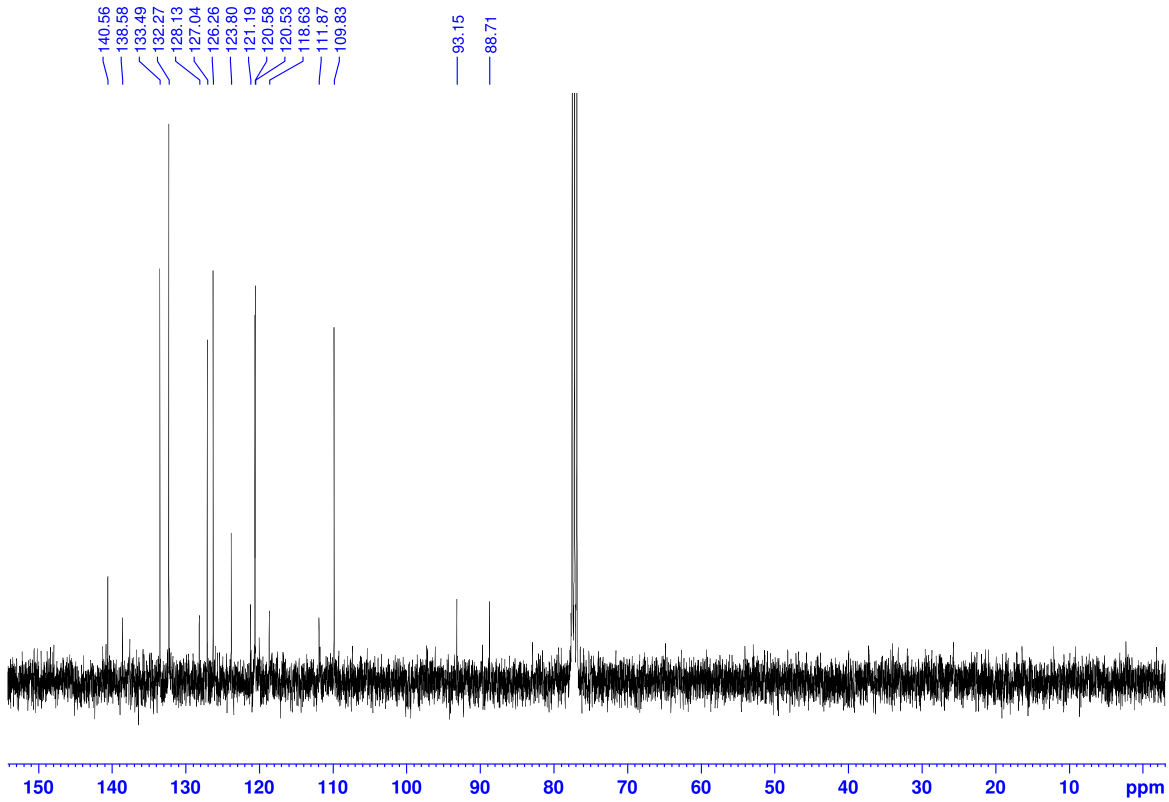

Figure S5. ^{13}C NMR spectrum of **CBZ-H** (100 MHz, $CDCl_3$).

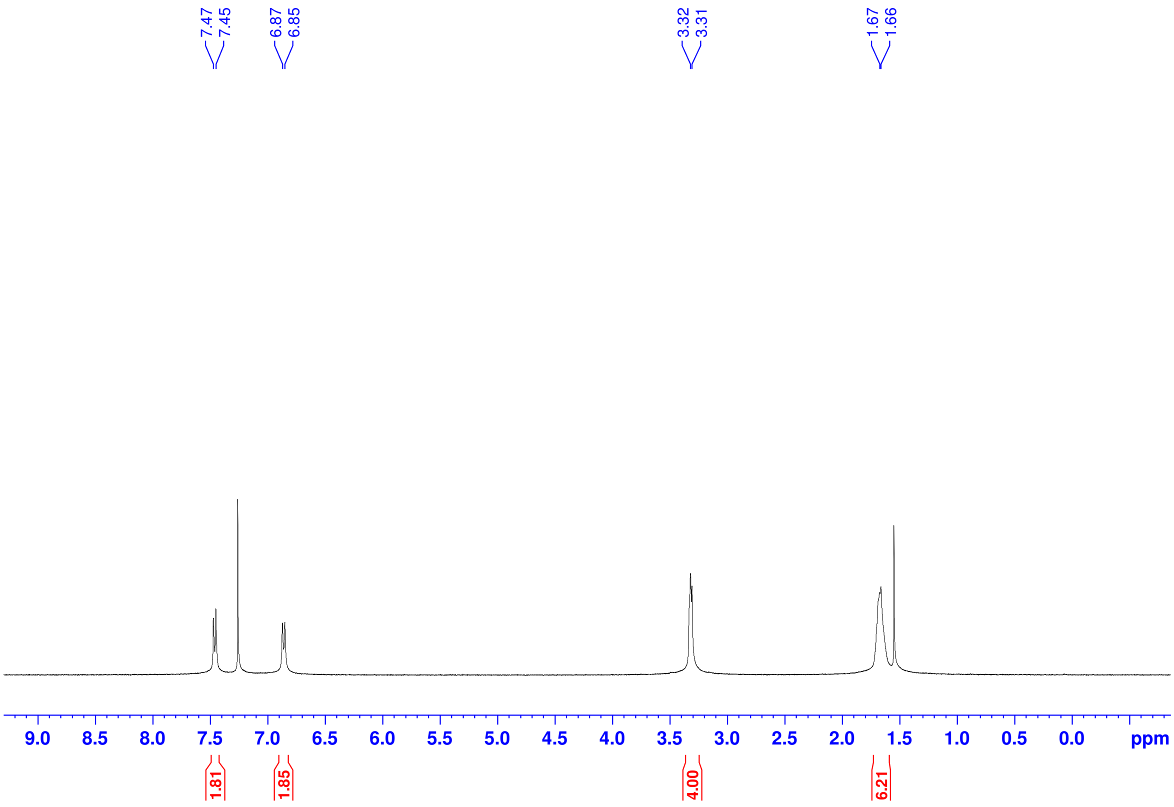

Figure S6. ¹H NMR spectrum of **PIP-F** (400 MHz, $CDCl_3$).

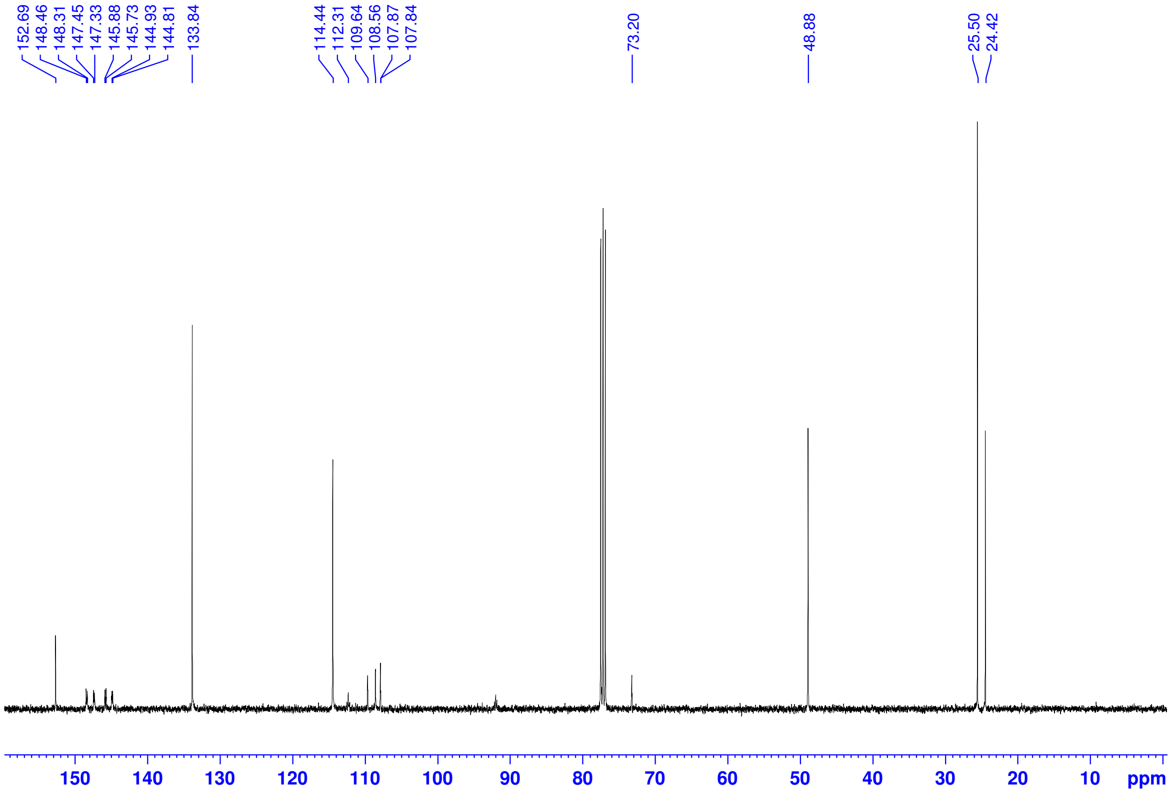

Figure S7. ¹³C NMR spectrum of **PIP-F** (100 MHz, $CDCl_3$).

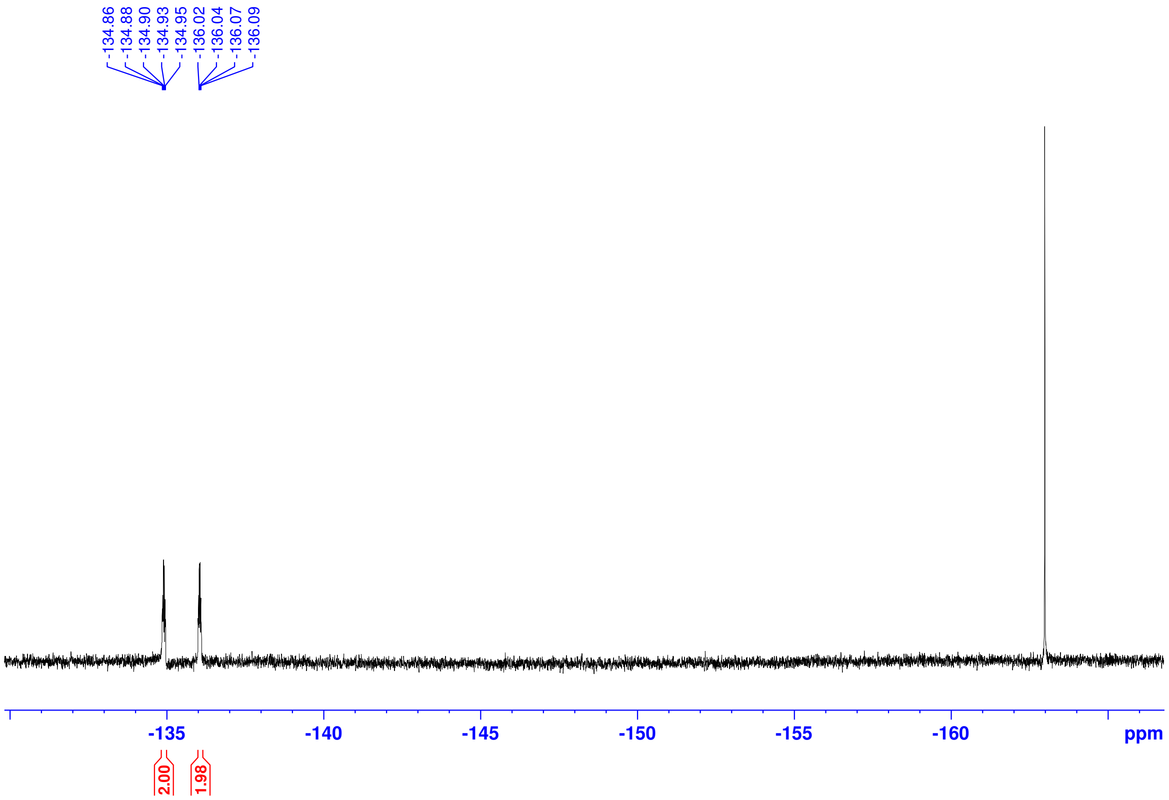

Figure S8. ^{19}F NMR spectrum of **PIP-F** (376 MHz, $CDCl_3$, hexafluorobenzene δ_F = –163 ppm).

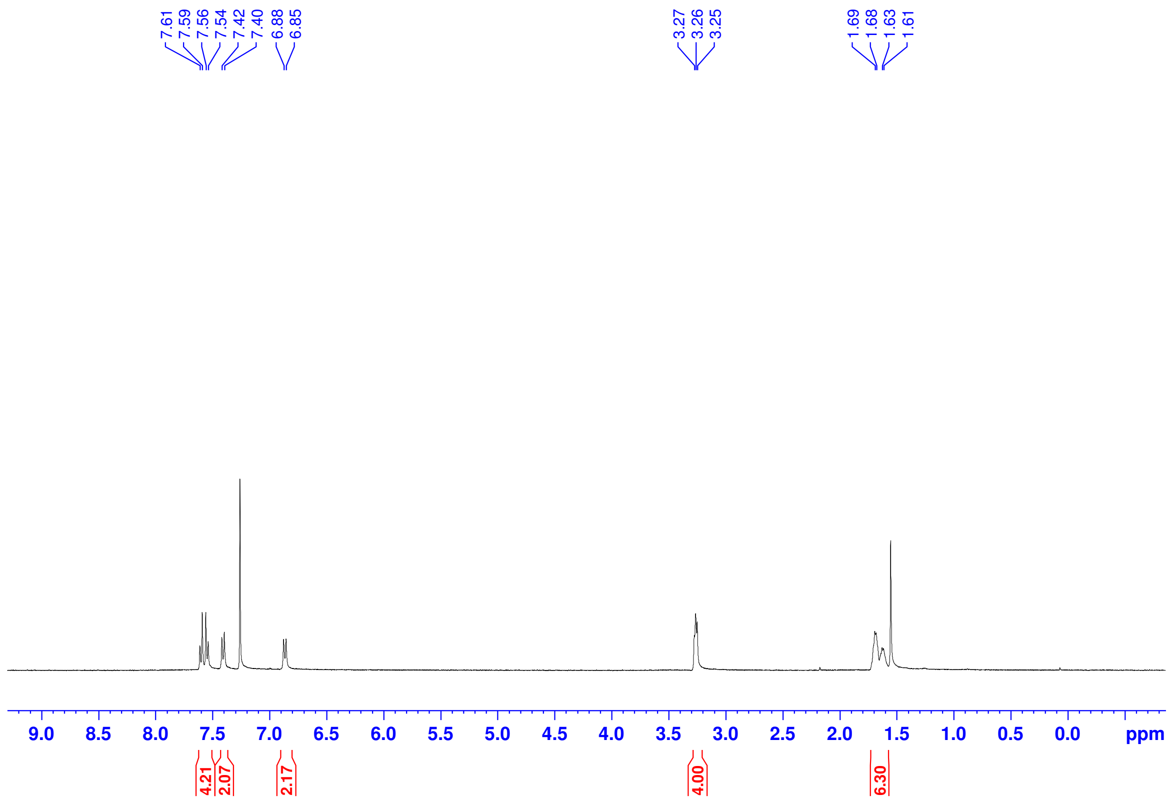

Figure S9. ^{1}H NMR spectrum of **PIP-H** (400 MHz, $CDCl_3$).

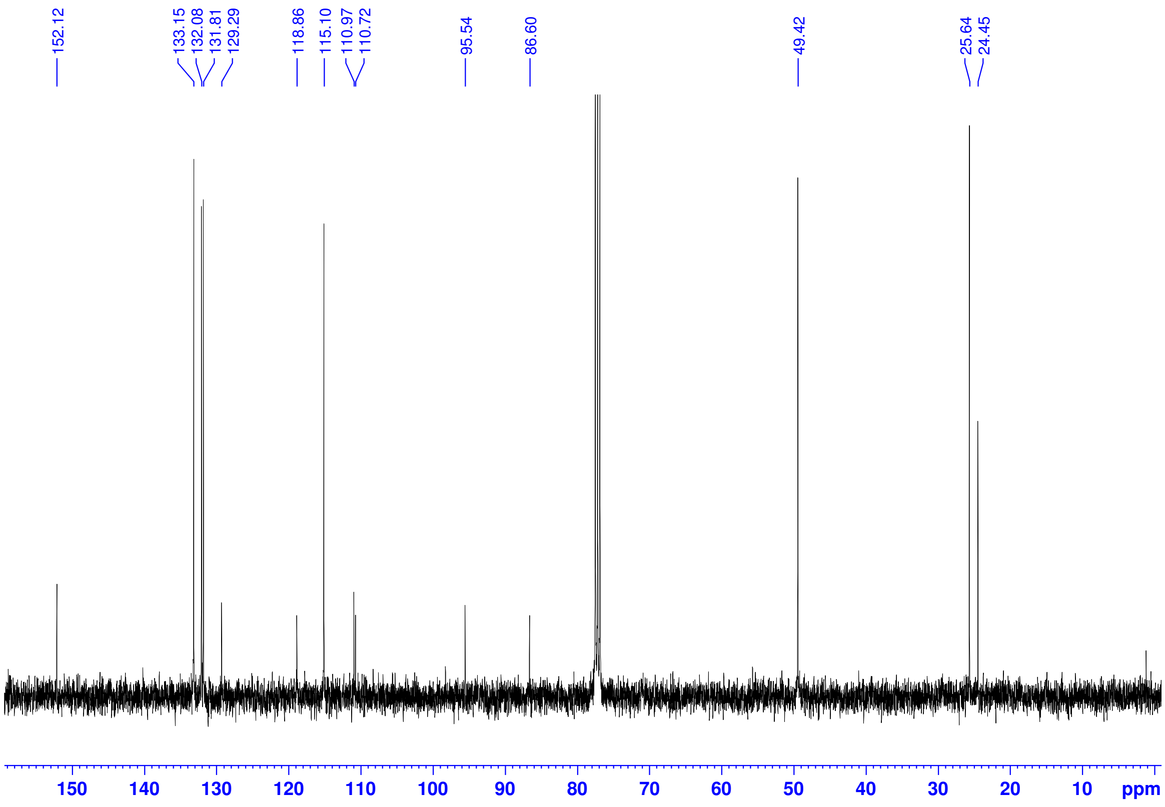

Figure S10. ^{13}C NMR spectrum of **PIP-H** (100 MHz, $CDCl_3$).

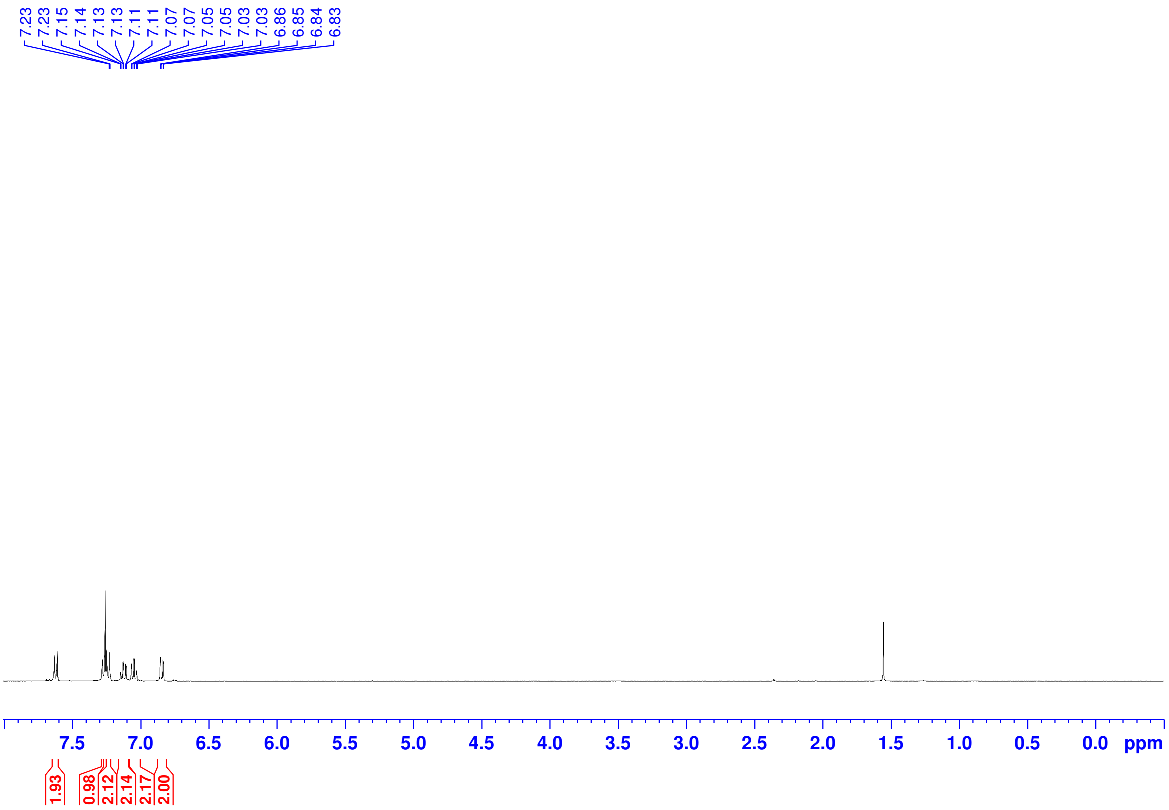

Figure S11. ^{1}H NMR spectrum of **PTZ-F** (400 MHz, $CDCl_3$).

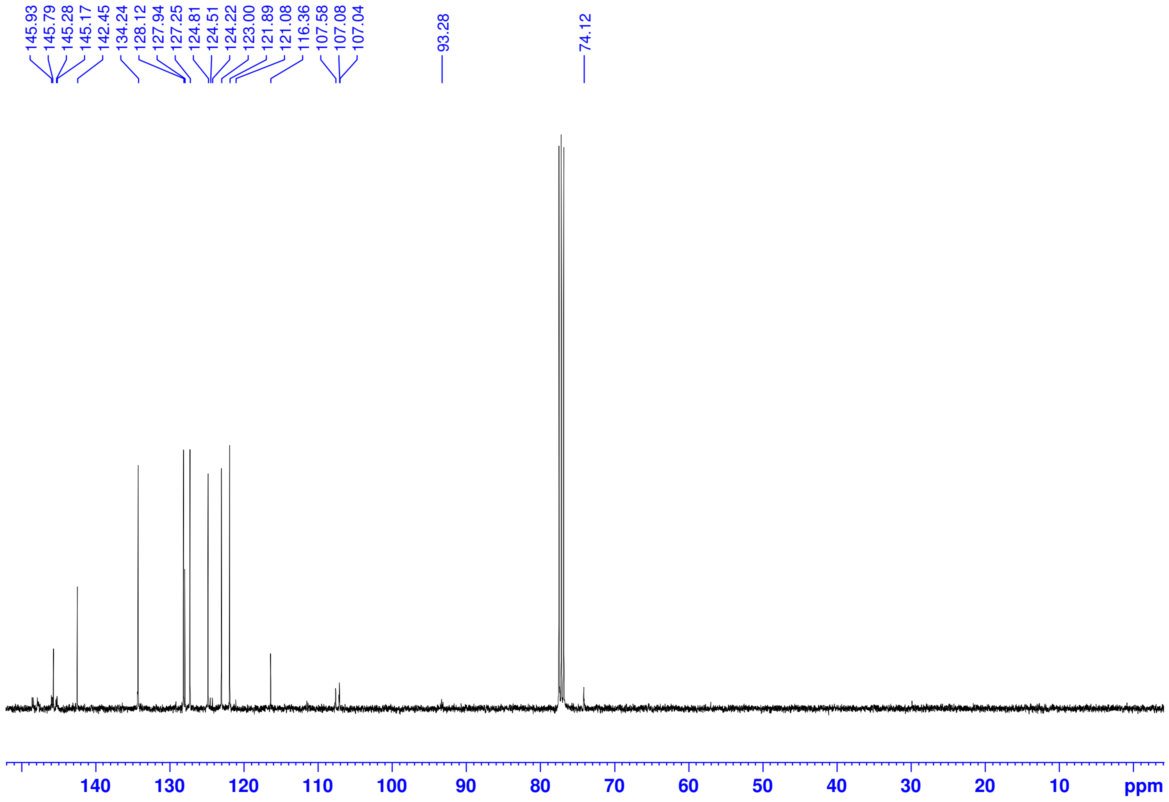

Figure S12. ^{13}C NMR spectrum of **PTZ-F** (100 MHz, $CDCl_3$).

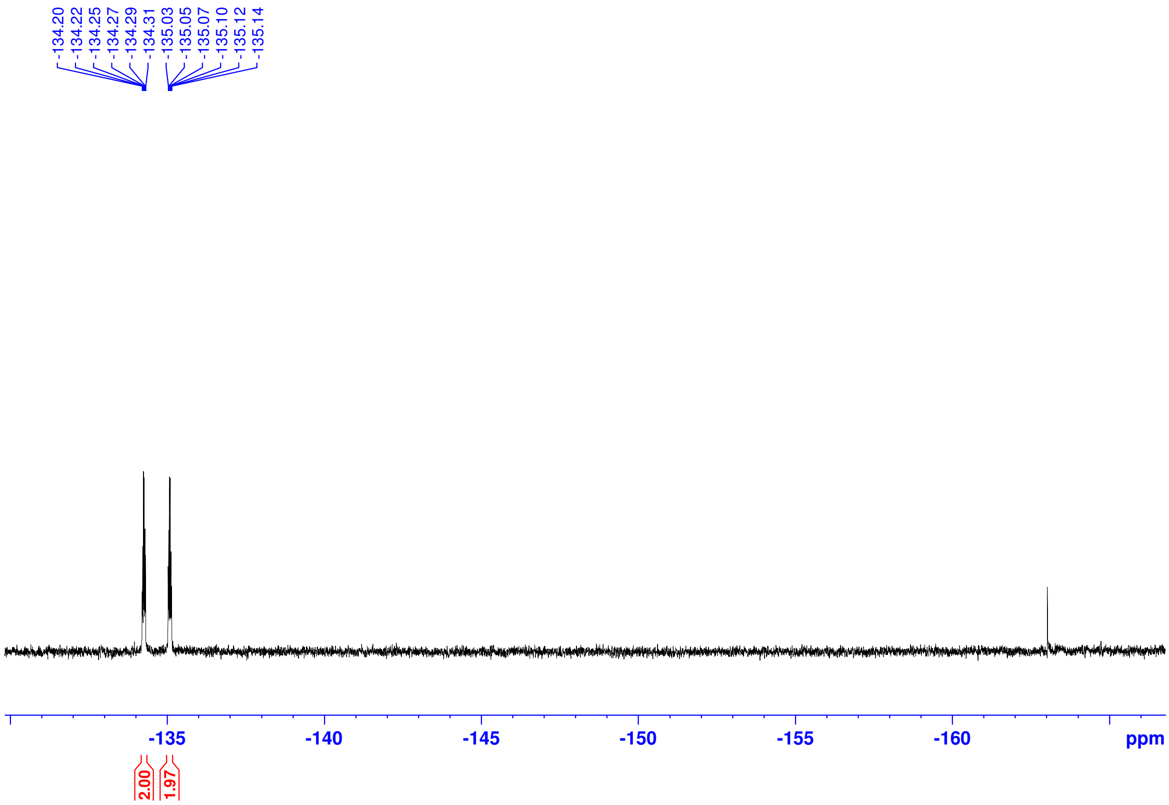

Figure S13. ^{19}F NMR spectrum of **PTZ-F** (376 MHz, $CDCl_3$, hexafluorobenzene δ_F = –163 ppm).

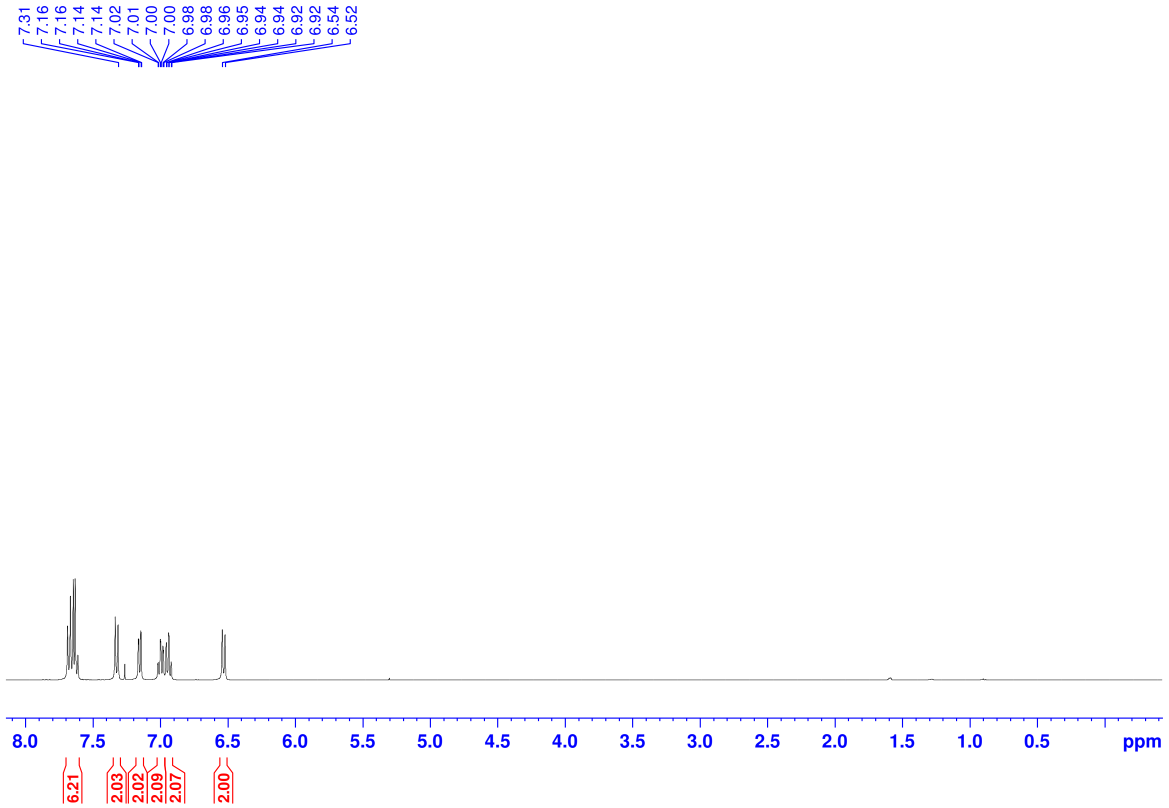

Figure S14. ^{1}H NMR spectrum of **PTZ-H** (400 MHz, $CDCl_3$).

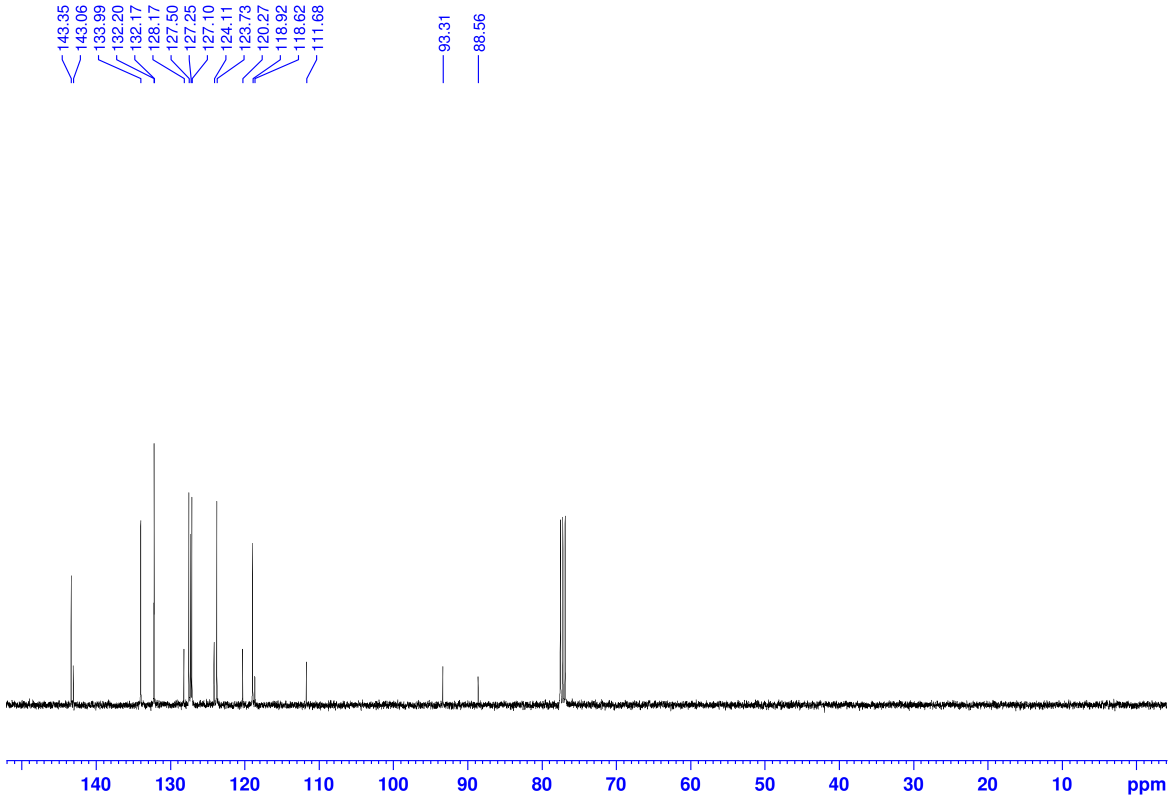

Figure S15. ^{13}C NMR spectrum of **PTZ-H** (100 MHz, $CDCl_3$).

3. X-ray Crystallographic Analysis

Table S1. Crystallographic data of **CBZ-H**, **PIP-F**, **PTZ-F**, **PTZ-H**.

	**CBZ-H**	**PIP-F**	**PTZ-F**	**PTZ-H**
CCDC #	2151066	2151065	215108	2151067
Empirical Formula	$C_{27}H_{16}N_2$	$C_{20}H_{14}F_4N_2$	$C_{27}H_{12}F_4N_2S$	$C_{27}H_{16}N_2$
Formula weight	368.42	358.33	472.45	400.48
Temperature [K]	298	297	298	100
Crystal Color / Habit	Colourless / block	Yellow / block	Orange / Plate	Yellow / block
Crystal Size [mm]	0.763 x 0.653 x 0.591	0.42 x 0.375 x 0.27	0.501 x 0.323 x 0.201	0.857 x 0.71 x 0.663
Crystal System	Monoclinic	Triclinic	Monoclinic	Triclinic
Space Group	P 1 21/c 1	P-1	C 1 2/c 1	P-1
a [Å]	8,3951(4)	6.3026(3)	8.464(2)	9.4462(2)
b [Å]	27.9304(11)	7.5073(3)	25.149(5)	11.9186(2)
c [Å]	8.7047(5)	18.3949(7)	10.370 (3)	19.0135(4)
α [°]	90	99.553(3)	90	83.182(2)
β [°]	106.182(5)	90.722(4)	104.500(5)	76.177(2)
γ [°]	90	94.046(4)	90	79.585(2)
V [Å^3]	1960.20(17)	855.89(6)	2137.2(8)	2038.00(7)
Z	4	2	4	4
R [$F^2 > 2s(F^2)$] [a]	0.0444	0.0529	0.0508	0.0362
wR_2 (F^2) [b]	0.1162	0.1739	0.1478	0.0962

[a] $R = \Sigma||F_o| - |F_c|| / \Sigma|F_o|$. [b] $wR = \{[\Sigma w(|F_o| - |F_c|)]/\Sigma w|F_o|\}^{1/2}$.

4. DFT calculation

Table S2. Dipole moment (debye) of all derivatives at franck-condon state.

	Dipole moment (debye)
**CBZ-F**	X = 0.0000 / Y = 0.0000 / Z = -27.3303 Tot = 27.3303
**CBZ-H**	X= 0.0000 / Y = 0.0000 / Z = -19.4571 Tot = 19.4571
**PIP-F**	X = -28.6317 / Y = -0.1896 / Z = 0.5683 Tot = 28.6379
**PIP-H**	X = -25.9340 / Y = 0.1709 / Z = -0.5371 Tot = 25.9401
**PTZ-F**	X = -37.1484 / Y = 0.0000 / Z = -1.3418 Tot = 37.1726
**PTZ-H**	X = 29.1970 / Y = 0.0000 / Z = -1.3936 Tot = 29.2302

Table S3. Cartesian coordinate for **CBZ-F** at the optimized geometry in S_0 state.

No.	Atom	Type	Coordinates (Angstroms)		
			X	Y	Z
1	6	0	-6.325002	-1.009219	0.648346
2	6	0	-4.942223	-1.007255	0.647257
3	6	0	-4.216086	0	0
4	6	0	-4.942223	1.007255	-0.647257
5	6	0	-6.325002	1.009219	-0.648346
6	6	0	-7.040324	0	0
7	6	0	-2.796471	0	0
8	9	0	-4.29625	1.986908	-1.27572
9	9	0	-6.977627	1.983993	-1.273584
10	9	0	-6.977627	-1.983993	1.273584
11	9	0	-4.29625	-1.986908	1.27572
12	6	0	-0.154951	0	0
13	6	0	0.55094	-0.996546	0.692781
14	6	0	0.55094	0.996546	-0.692781
15	6	0	1.938897	-0.990548	0.699584
16	1	0	0.006169	-1.762129	1.234675
17	6	0	1.938897	0.990548	-0.699584
18	1	0	0.006169	1.762129	-1.234675
19	6	0	2.636173	0	0
20	1	0	2.488065	-1.74289	1.256244
21	1	0	2.488065	1.74289	-1.256244
22	6	0	-1.584369	0	0
23	6	0	-8.470498	0	0
24	7	0	-9.627056	0	0
25	6	0	4.862922	1.087669	0.316647
26	6	0	4.862922	-1.087669	-0.316647
27	6	0	4.503792	2.372292	0.734615
28	6	0	6.215361	0.695691	0.203416
29	6	0	4.503792	-2.372292	-0.734615
30	6	0	6.215361	-0.695691	-0.203416
31	6	0	5.531504	3.266336	1.01484
32	1	0	3.465308	2.665456	0.844371
33	6	0	7.230947	1.612967	0.491131
34	6	0	5.531504	-3.266336	-1.01484
35	1	0	3.465308	-2.665456	-0.844371
36	6	0	7.230947	-1.612967	-0.491131
37	6	0	6.882416	2.897613	0.890871
38	1	0	5.280976	4.271304	1.339318
39	1	0	8.274083	1.322589	0.407523
40	6	0	6.882416	-2.897613	-0.890871
41	1	0	5.280976	-4.271304	-1.339318
42	1	0	8.274083	-1.322589	-0.407523
43	1	0	7.65736	3.62258	1.116393
44	1	0	7.65736	-3.62258	-1.116393
45	7	0	4.04854	0	0

Table S4. Cartesian coordinate for **CBZ-H** at the optimized geometry in S_0 state.

No.	Atom	Type	Coordinates (Angstroms)		
			X	Y	Z
1	6	0	-3.01277	0.347214	-5.942111
2	6	0	-3.396685	0.400768	-4.590574
3	6	0	-2.465286	0.288705	-3.564068
4	6	0	-1.126064	0.114147	-3.924988
5	6	0	-0.721042	0.073694	-5.278095
6	6	0	-1.67717	0.188847	-6.292427
7	1	0	-3.76772	0.436392	-6.716162
8	1	0	-4.443951	0.535259	-4.339153
9	1	0	-2.771223	0.340627	-2.524681
10	1	0	-1.37822	0.158907	-7.336067
11	6	0	1.126064	-0.114147	-3.924988
12	6	0	2.465286	-0.288705	-3.564068
13	6	0	3.396685	-0.400768	-4.590574
14	6	0	3.01277	-0.347214	-5.942111
15	6	0	1.67717	-0.188847	-6.292427
16	6	0	0.721042	-0.073694	-5.278095
17	1	0	2.771223	-0.340627	-2.524681
18	1	0	4.443951	-0.535259	-4.339153
19	1	0	3.76772	-0.436392	-6.716162
20	1	0	1.37822	-0.158907	-7.336067
21	7	0	0	0	-3.111426
22	6	0	0	0	-1.697023
23	6	0	-0.825617	-0.886699	-0.999403
24	6	0	0.825617	0.886699	-0.999403
25	6	0	-0.832644	-0.88045	0.3893
26	1	0	-1.449757	-1.584456	-1.548507
27	6	0	0.832644	0.88045	0.3893
28	1	0	1.449757	1.584456	-1.548507
29	6	0	0	0	1.098643
30	1	0	-1.472512	-1.567174	0.933035
31	1	0	1.472512	1.567174	0.933035
32	6	0	0	0	2.530448
33	6	0	0	0	3.743746
34	6	0	0	0	5.174825
35	6	0	-0.841527	-0.876297	5.880753
36	6	0	0.841527	0.876297	5.880753
37	6	0	-0.843185	-0.877966	7.267964
38	1	0	-1.490184	-1.551808	5.334316
39	6	0	0.843185	0.877966	7.267964
40	1	0	1.490184	1.551808	5.334316
41	6	0	0	0	7.961301
42	1	0	-1.491441	-1.553038	7.815425
43	1	0	1.491441	1.553038	7.815425
44	6	0	0	0	9.400583
45	7	0	0	0	10.558887

Table S5. Cartesian coordinate for **PIP-F** at the optimized geometry in S_0 state.

No.	Atom	Type	Coordinates (Angstroms)		
			X	Y	Z
1	6	0	-7.517135	-1.077746	-0.618561
2	6	0	-6.055852	1.053701	0.674743
3	6	0	-7.484705	1.278818	0.194916
4	6	0	-8.265659	-0.033497	0.20611
5	1	0	-7.499275	-0.769414	-1.671158
6	1	0	-8.017856	-2.049868	-0.569467
7	1	0	-6.069911	0.725386	1.729597
8	1	0	-5.496323	1.986784	0.632243
9	1	0	-7.959621	2.024437	0.84048
10	1	0	-7.459247	1.687916	-0.822661
11	1	0	-8.36565	-0.387286	1.241388
12	1	0	-9.277314	0.112771	-0.184643
13	6	0	-6.078087	-1.247754	-0.135963
14	1	0	-5.556278	-1.930762	-0.809517
15	1	0	-6.072061	-1.696803	0.874212
16	6	0	-3.979061	0.006281	-0.075767
17	6	0	-3.251951	-1.193203	0.07488
18	6	0	-3.241687	1.205642	-0.212742
19	6	0	-1.863802	-1.193176	0.081409
20	1	0	-3.765713	-2.136335	0.208937
21	6	0	-1.860094	1.204299	-0.198644
22	1	0	-3.759416	2.145031	-0.367267
23	6	0	-1.142974	0.001812	-0.051522
24	1	0	-1.328854	-2.129376	0.206148
25	1	0	-1.319395	2.137817	-0.317672
26	6	0	0.281481	0.000442	-0.039176
27	6	0	1.495664	0.001375	-0.027699
28	6	0	2.912724	0.000962	-0.013131
29	6	0	3.640869	-1.195026	0.041855
30	6	0	3.642846	1.196357	-0.052251
31	6	0	5.022887	-1.197905	0.056662
32	6	0	5.024857	1.198067	-0.036698
33	6	0	5.740979	-0.00021	0.017879
34	7	0	-5.371361	0.031981	-0.132139
35	6	0	7.170368	-0.000747	0.033646
36	7	0	8.327089	-0.001213	0.046426
37	9	0	2.998153	2.360976	-0.104582
38	9	0	5.678389	2.355829	-0.073419
39	9	0	5.674461	-2.356232	0.107891
40	9	0	2.994198	-2.359089	0.080076

Table S6. Cartesian coordinate for **PIP-H** at the optimized geometry in S_0 state.

No.	Atom	Type	Coordinates (Angstroms)		
			X	Y	Z
1	6	0	-5.659684	1.053212	1.227757
2	6	0	-5.374555	1.276221	-0.26085
3	6	0	-5.372088	-1.084007	-0.70431
4	6	0	-5.657182	-1.433762	0.759615
5	6	0	-6.398509	-0.27407	1.436245
6	1	0	-6.317721	1.319423	-0.818288
7	1	0	-4.865234	2.22215	-0.436118
8	1	0	-4.705021	1.040107	1.770484
9	1	0	-6.248433	1.888564	1.621143
10	1	0	-6.319539	-0.912073	-1.229104
11	1	0	-4.865742	-1.889519	-1.236909
12	1	0	-6.247003	-2.354672	0.814583
13	1	0	-4.707226	-1.620578	1.277105
14	1	0	-7.404115	-0.192793	1.000763
15	1	0	-6.528936	-0.474934	2.504221
16	7	0	-4.601232	0.156699	-0.810517
17	6	0	-3.225453	0.122398	-0.627524
18	6	0	-2.507703	-1.095623	-0.56748
19	6	0	-2.466874	1.315167	-0.544033
20	6	0	-1.126057	-1.115214	-0.454796
21	1	0	-3.025086	-2.045457	-0.596059
22	6	0	-1.08791	1.2877	-0.427553
23	1	0	-2.951647	2.281619	-0.588025
24	6	0	-0.383182	0.072802	-0.382166
25	1	0	-0.611067	-2.069933	-0.410226
26	1	0	-0.54058	2.223729	-0.373245
27	6	0	1.038999	0.048311	-0.26087
28	6	0	2.249731	0.027542	-0.157795
29	6	0	3.673466	0.003926	-0.036701
30	6	0	4.361638	-1.221749	0.009169
31	6	0	4.398541	1.206666	0.038098
32	6	0	5.743288	-1.24646	0.126799
33	1	0	3.803802	-2.149842	-0.04849
34	6	0	5.780228	1.186666	0.155768
35	1	0	3.869263	2.152419	0.002815
36	6	0	6.45415	-0.041107	0.200195
37	1	0	6.273941	-2.191572	0.16219
38	1	0	6.339445	2.114035	0.213431
39	6	0	7.887256	-0.064395	0.321609
40	7	0	9.041559	-0.083272	0.419468

Table S7. Cartesian coordinate for **PTZ-F** at the optimized geometry in S_0 state.

No.	Atom	Type	Coordinates (Angstroms)		
			X	Y	Z
1	6	0	-4.419673	-3.56341	-0.717872
2	6	0	-3.742406	-2.363864	-0.502946
3	6	0	-4.425555	-1.230246	-0.042475
4	6	0	-5.803565	-1.342225	0.208168
5	6	0	-6.481074	-2.532582	-0.046049
6	6	0	-5.791684	-3.654155	-0.503065
7	6	0	-5.803566	1.342223	0.208175
8	6	0	-4.425555	1.230246	-0.042468
9	6	0	-3.742407	2.363867	-0.502933
10	1	0	-2.675741	2.320027	-0.684965
11	6	0	-4.419675	3.563414	-0.717852
12	6	0	-5.791686	3.654157	-0.503045
13	6	0	-6.481075	2.532581	-0.046035
14	1	0	-3.861529	-4.426791	-1.064976
15	1	0	-2.67574	-2.320022	-0.684978
16	1	0	-7.549854	-2.58011	0.139955
17	1	0	-6.321463	-4.583483	-0.681271
18	1	0	-3.861531	4.426797	-1.064951
19	1	0	-6.321465	4.583486	-0.681246
20	1	0	-7.549855	2.580107	0.139969
21	7	0	-3.76276	0	0.198459
22	16	0	-6.665709	-0.000003	0.97962
23	6	0	-2.329554	0	0.145108
24	6	0	-1.618145	-0.000004	1.342712
25	6	0	-1.654138	0.000004	-1.07885
26	6	0	-0.226919	-0.000004	1.325254
27	1	0	-2.162925	-0.000007	2.281121
28	6	0	-0.266275	0.000004	-1.105155
29	1	0	-2.222587	0.000007	-2.004554
30	6	0	0.455838	0	0.100283
31	1	0	0.334112	-0.000006	2.253495
32	1	0	0.265585	0.000007	-2.050428
33	6	0	1.886614	0	0.0771
34	6	0	3.098077	0	0.05813
35	6	0	4.518134	0	0.038568
36	6	0	5.227734	0.000001	-1.168314
37	6	0	5.259273	0	1.226362
38	6	0	6.610517	0	-1.188728
39	6	0	6.642091	-0.000001	1.21045
40	6	0	7.34112	0	0.001474
41	6	0	8.771302	0	-0.017331
42	7	0	9.927706	0	-0.032659
43	9	0	4.62853	-0.000001	2.398222
44	9	0	7.309798	-0.000001	2.359608
45	9	0	7.247877	0.000001	-2.355031
46	9	0	4.566377	0.000001	-2.323207

Table S8. Cartesian coordinate for **PTZ-H** at the optimized geometry in S_0 state.

No.	Atom	Type	Coordinates (Angstroms)		
			X	Y	Z
1	6	0	3.511218	3.567265	-0.700187
2	6	0	2.83241	2.367952	-0.489132
3	6	0	3.514764	1.230294	-0.037007
4	6	0	4.893709	1.339196	0.210681
5	6	0	5.572728	2.529483	-0.039957
6	6	0	4.884141	3.654594	-0.489441
7	6	0	4.88958	-1.345151	0.198925
8	6	0	3.511179	-1.229322	-0.048378
9	6	0	2.825238	-2.360122	-0.512125
10	1	0	1.758297	-2.312808	-0.691702
11	6	0	3.499978	-3.559822	-0.7337
12	6	0	4.872374	-3.653977	-0.522354
13	6	0	5.564612	-2.535459	-0.062149
14	1	0	2.95349	4.433408	-1.041114
15	1	0	1.765112	2.326358	-0.667816
16	1	0	6.642147	2.574143	0.143166
17	1	0	5.415078	4.58381	-0.664822
18	1	0	2.939569	-4.420762	-1.083295
19	1	0	5.400155	-4.583449	-0.705726
20	1	0	6.633756	-2.585532	0.121153
21	7	0	2.850255	0.000361	0.19793
22	16	0	5.755622	-0.007698	0.974337
23	6	0	1.416349	0.001726	0.147358
24	6	0	0.705631	-0.010972	1.344988
25	6	0	0.737776	0.013644	-1.074455
26	6	0	-0.686037	-0.011711	1.328831
27	1	0	1.251142	-0.020579	2.283116
28	6	0	-0.650489	0.013688	-1.097613
29	1	0	1.30409	0.022807	-2.001608
30	6	0	-1.37475	0.000825	0.106899
31	1	0	-1.244476	-0.02179	2.2587
32	1	0	-1.182754	0.023175	-2.042737
33	6	0	-2.807297	0.000458	0.085385
34	6	0	-4.020147	0.00026	0.06715
35	6	0	-5.451416	0.000193	0.047944
36	6	0	-6.141125	0.043818	-1.175296
37	6	0	-6.172356	-0.043439	1.253029
38	6	0	-7.528271	0.0441	-1.195473
39	6	0	-7.559524	-0.043419	1.237634
40	6	0	-8.23679	0.000481	0.012189
41	6	0	-9.675985	0.000856	-0.006205
42	7	0	-10.834153	0.001176	-0.021042
43	1	0	-5.637927	-0.077348	2.195858
44	1	0	-8.119074	-0.077177	2.165805
45	1	0	-8.063692	0.077907	-2.137777
46	1	0	-5.582591	0.077604	-2.104057

5. Photophysical Characteristics

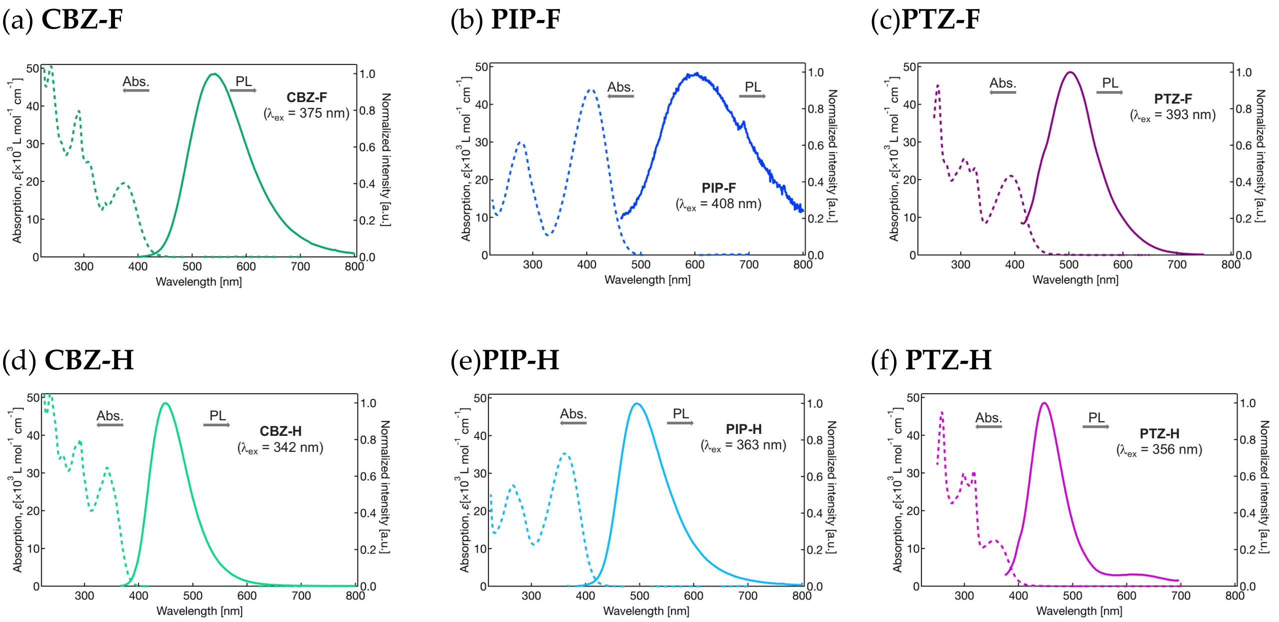

Figure S16. UV-vis and PL spectra of all derivatives in CH_2Cl_2 solution. Concentration: 1.0×10^{-5} mol L^{-1} for UV-vis and PL.

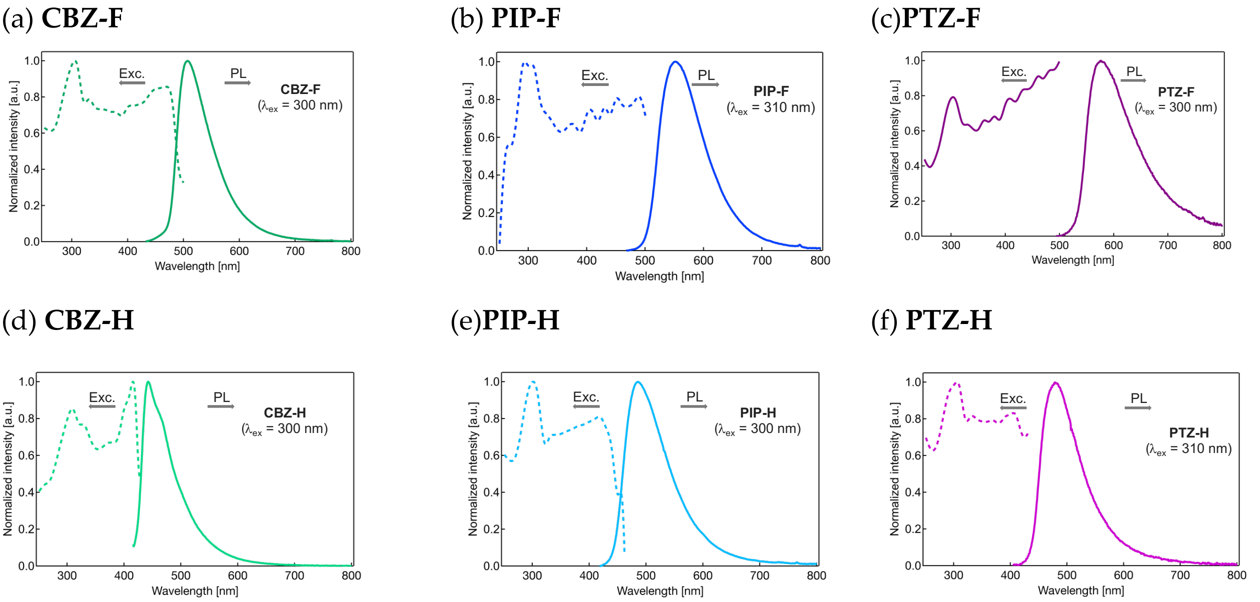

Figure S17. Excitation and PL spectra of all derivatives in crystalline state. Excitation spectra were obtained by monitoring PL at the maximum wavelength.

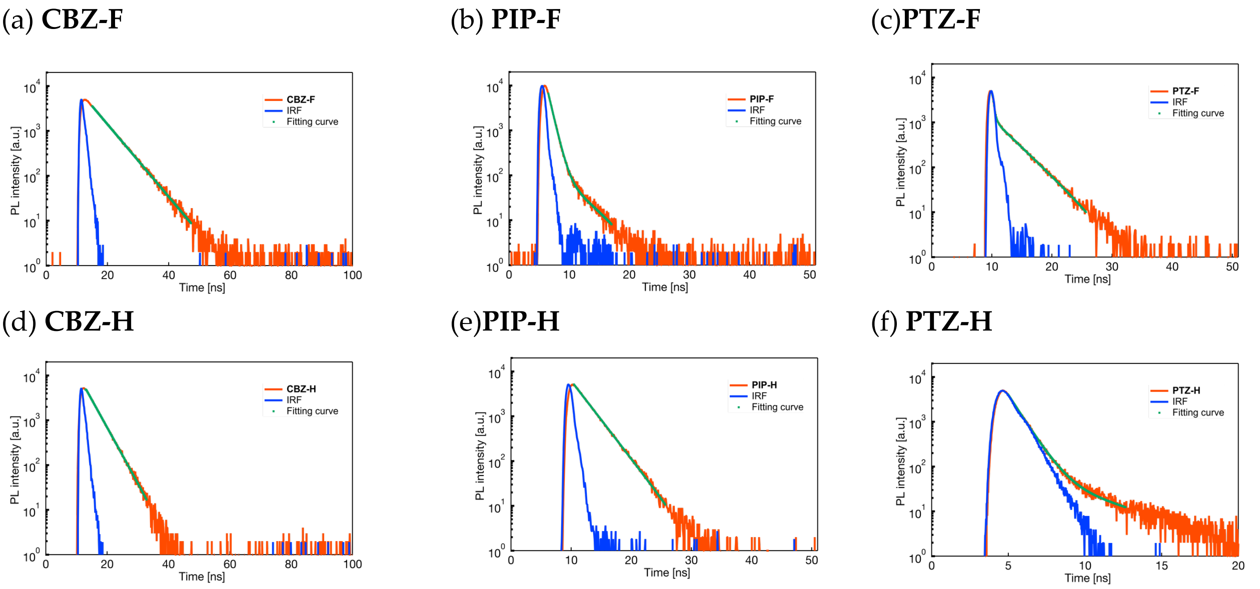

Figure S18. PL decay curve of all derivatives in CH_2Cl_2 solution. They were measured by monitoring PL at maximum wavelength (λ_{PL} = 542 nm for **CBZ-F**, 447 nm for **CBZ-H**, 603 nm for **PIP-F**, 496 nm for **PIP-H**, 501 nm for **PTZ-F**, 447 nm for **PTZ-H**). Concentration: 1.0×10^{-5} mol L^{-1}.

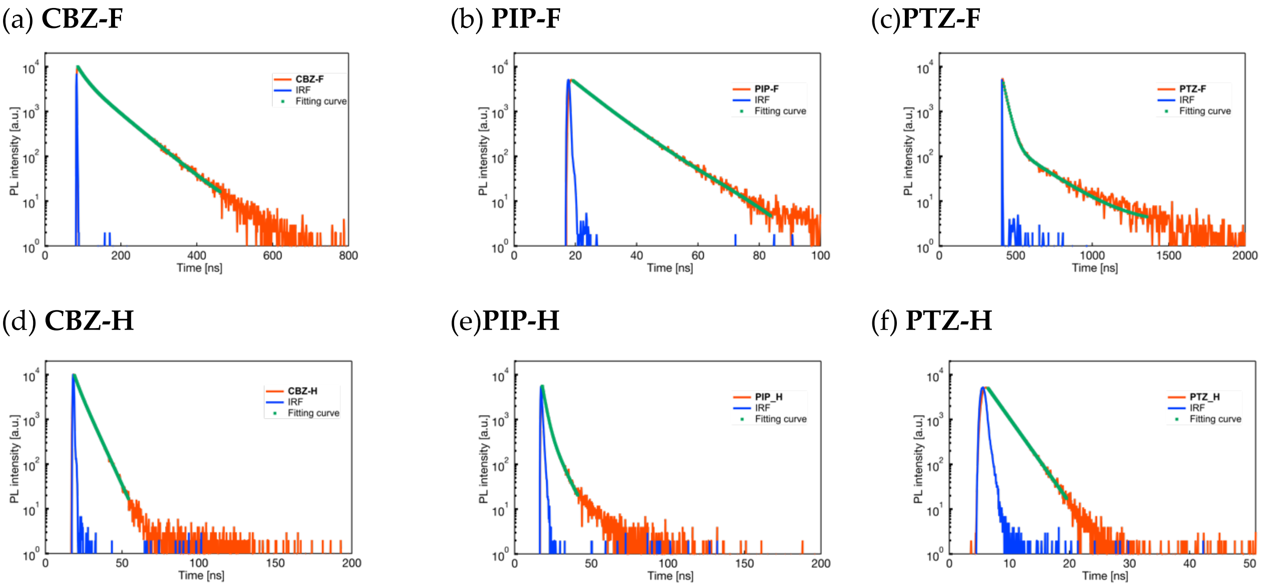

Figure S19. PL decay curve of all derivatives in crystalline state. They were measured by monitoring PL at maximum wavelength (λ_{PL} = 507 nm for **CBZ-F**, 442 nm for **CBZ-H**, 551 nm for **PIP-F**, 485 nm for **PIP-H**, 575 nm for **PTZ-F**, 478 nm for **PTZ-H**).

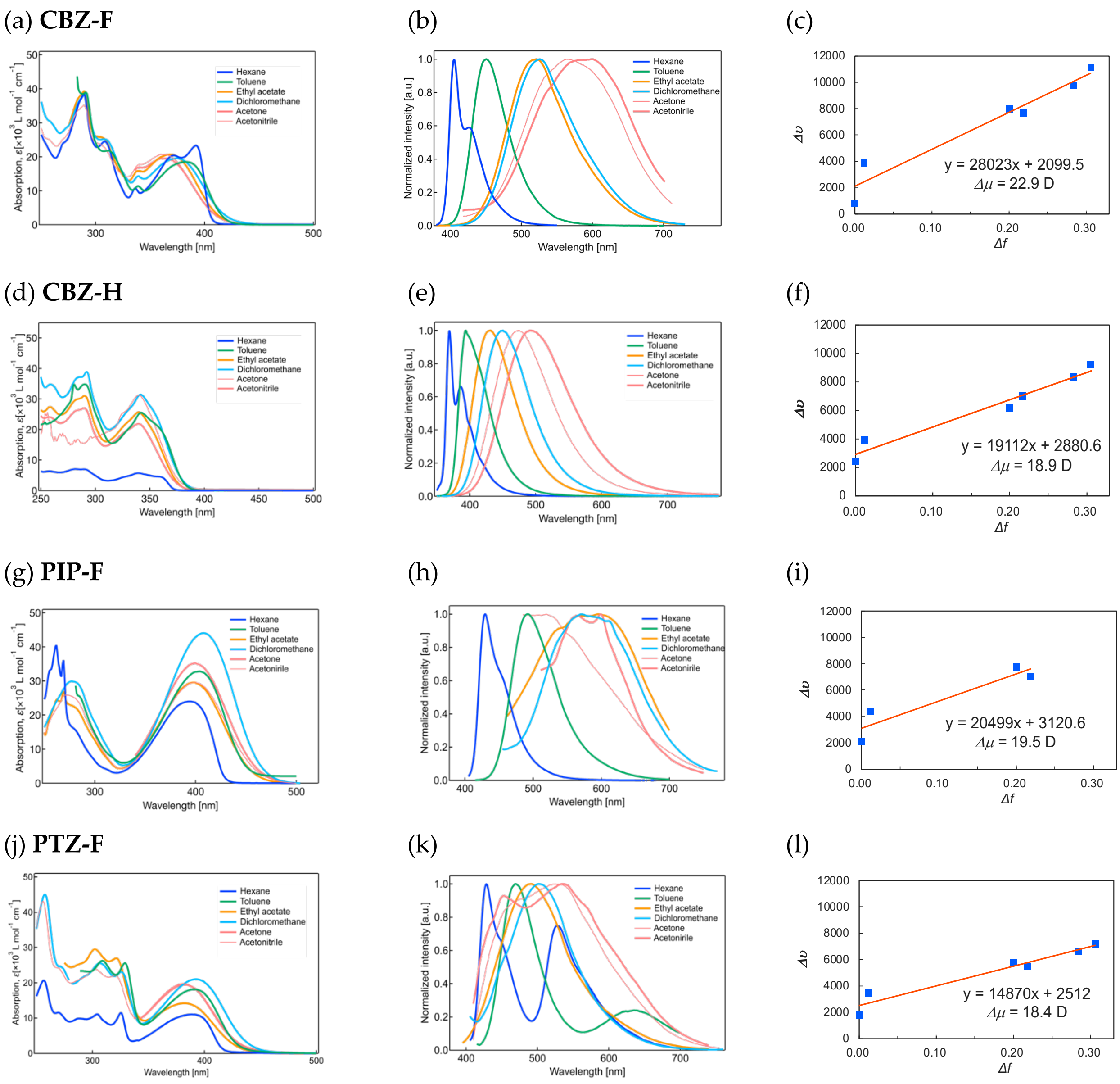

Figure S20. UV-vis and PL spectra of **CBZ-F**, **CBZ-H**, **PIP-F**, **PTZ-F** in various solvents. Concentration: 1.0×10^{-5} mol L^{-1} for UV-vis and PL. Lippert-Mataga plot of **CBZ-F**, **CBZ-H**, **PIP-F**, **PTZ-F.**

Table S9. Photophysical properties of **CBZ-F** in various solvents.

Solvent	λ_{abs} [nm]	λ_{PL} [nm]	Φ_{PL}
Hexane	392	405	0.85
Toluene	383	450	0.92
Ethyl acetate	368	521	0.55
Dichloromethane	375	526	0.65
Acetone	364	564	0.03
Acetonitrile	360	580	0.01

Table S10. Photophysical properties of **CBZ-H** in various solvents.

Solvent	λ_{abs} [nm]	λ_{PL} [nm]	Φ_{PL}
Hexane	339	369	0.8
Toluene	342	394	0.95
Ethyl acetate	340	430	0.99
Dichloromethane	341	447	1.0
Acetone	340	473	0.77
Acetonitrile	340	494	0.82

Table S11. Photophysical properties of **PIP-F** in various solvents.

Solvent	λ_{abs} [nm]	λ_{PL} [nm]	Φ_{PL}
Hexane	394	430	0.47
Toluene	404	491	0.37
Ethyl acetate	398	577	0.01
Dichloromethane	408	572	0.02
Acetone	399	-	<0.01
Acetonitrile	400	-	<0.01

Table S12. Photophysical properties of **PTZ-F** in various solvents.

Solvent	λ_{abs} [nm]	λ_{PL} [nm]	Φ_{PL}
Hexane	389	418	0.06
Toluene	391	452	0.03
Ethyl acetate	382	490	<0.01
Dichloromethane	393	501	<0.01
Acetone	382	511	<0.01
Acetonitrile	381	524	<0.01

Table S13. Photophysical properties of **CBZ-F** in THF/H_2O mixed solution.

Water ration [%]	PL intensity [a.u.]	Quantum yield [-]	Peak wavelength [nm]
0	1158	0.63	542
10	145	0.11	572
20	70	0.06	580
30	47	0.04	577
40	33	0.03	580
50	22	0.02	588
60	22	0.02	588
70	713	0.36	481
75	697	0.44	550
80	568	0.42	543
85	1179	0.59	534
90	948	0.50	527

Table S14. Photophysical properties of **PIP-F** in THF/H_2O mixed solution.

Water ration [%]	PL intensity [a.u.]	Quantum yield [-]	Peak wavelength [nm]
0	8.74	0.01	n.d.
10	7.93	< 0.01	n.d.
20	8.55	< 0.01	n.d.
30	7.56	< 0.01	n.d.
40	6.96	< 0.01	n.d.
50	8.09	< 0.01	n.d.
60	7.33	< 0.01	n.d.
70	61.4	0.06	551
75	66.1	0.07	552
80	73.6	0.08	548
85	61.1	0.07	554
90	57.1	0.06	556

(a) **CBZ-F**

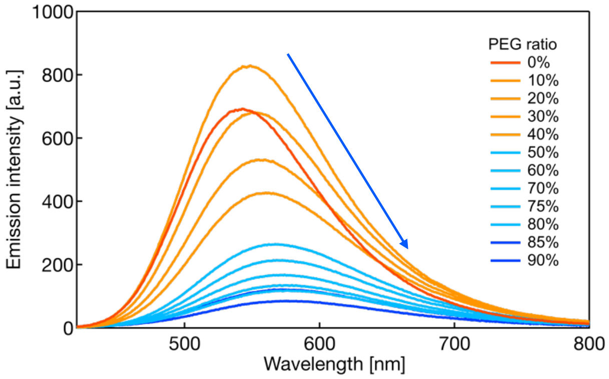

(b) **PIP-F**

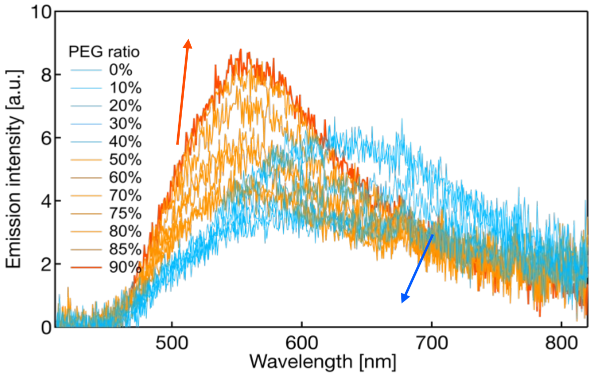

Figure S21. PL spectra of (a) **CBZ-F** and (b) **PIP-F** in THF/polyethylene glycol (PEG) mixed solution.

Table S15. Photophysical properties of **CBZ-F** in THF/PEG mixed solution.

PEG ratio [%]	PL intensity [a.u.]	Quantum yield [-]	Peak wavelength [nm]
0	695	0.61	543
10	829	0.50	548
20	681	0.42	552
30	532	0.35	558
40	428	0.29	561
50	265	0.20	567
60	214	0.17	568
70	168	0.14	573
75	136	0.13	571
80	120	0.12	576
85	123	0.13	571
90	87	0.11	575

Table S16. Photophysical properties of **PIP-F** in THF/PEG mixed solution.

PEG ratio [%]	PL intensity [a.u.]	Quantum yield [-]	Peak wavelength [nm]
0	7.0	0.011	625
10	6.6	0.009	677
20	5.1	0.007	682
30	4.3	0.006	683
40	5.1	0.006	599
50	5.1	0.006	548
60	5.1	0.007	580
70	6.2	0.008	536
75	6.5	0.009	562
80	8.1	0.010	550
85	9.0	0.010	549
90	9.7	0.012	552

5. Dynamic light-scattering measurement

The particle size of nanocrystal and microcrystals formed in THF/H_2O solvent solution was measured using an ELSZ-1000 (Otsuka Electronics, Co., Ltd.).

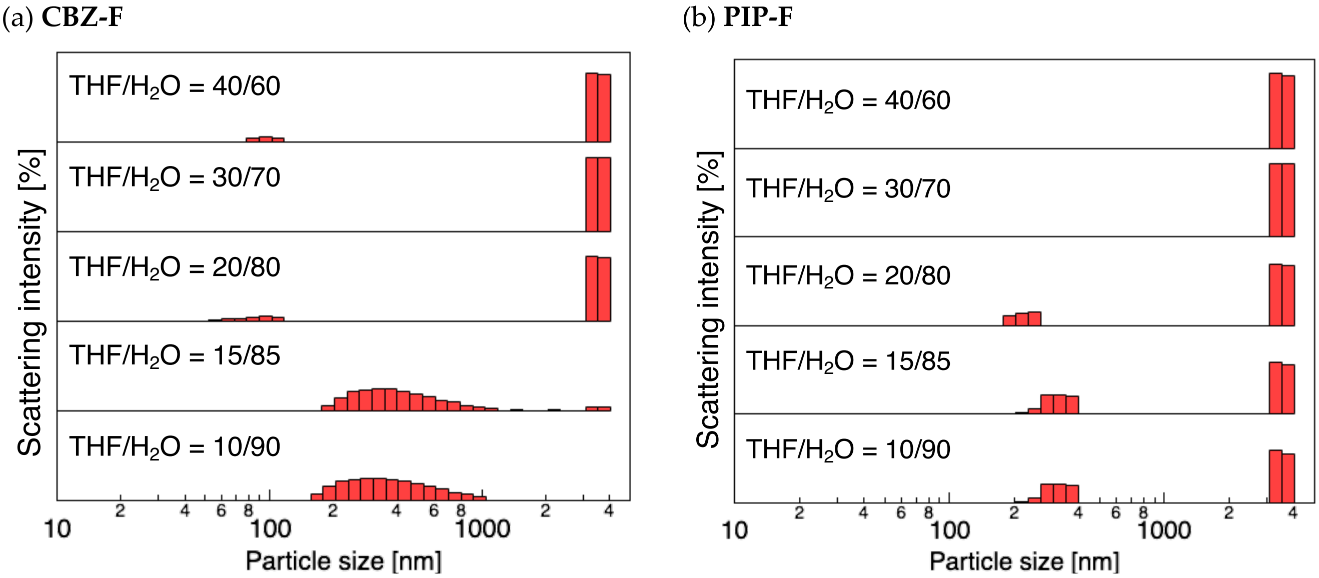

Figure S22. DLS profiles of (a) **CBZ-F** and (b) **PIP-F** in THF/H_2O mixed solution.
